# π-Electron systems containing Si=Si double bonds

**DOI:** 10.1080/14686996.2017.1414552

**Published:** 2018-02-07

**Authors:** Tsukasa Matsuo, Naoki Hayakawa

**Affiliations:** ^a^ Department of Applied Chemistry, Faculty of Science and Engineering, Kindai University, Osaka, Japan

**Keywords:** Silicon, π-electron systems, disilenes, conjugation, bulky protecting groups, 20 Organic and soft materials (colloids, liquid crystals, gel, polymers), 104 Carbon and related materials

## Abstract

Sterically large substituents can provide kinetic stabilization to various types of low-coordinate compounds. For example, regarding the chemistry of the group 14 elements, since West et al. introduced the concept of kinetic protection of the otherwise highly reactive Si=Si double bond by bulky mesityl (2,4,6-trimethylphenyl) groups in 1981, a number of unsaturated compounds of silicon and its group homologs have been successfully isolated by steric effects using the appropriate large substituents. However, the functions and applications of the Si–Si π-bonds consisting of the 3*p*π electrons on the formally *sp* ^2^-hybridized silicon atoms have rarely been explored until 10 years ago, when Scheschkewitz and Tamao independently reported the model systems of the oligo(*p*-phenylenedisilenylene)s (Si–OPVs) in 2007. This review focuses on the recent advances in the chemistry of π-electron systems containing Si=Si double bonds, mainly published in the last decade. The synthesis, characterization, and potential application of a variety of donor-free π-conjugated disilene compounds are described.

## Graphical abstract

**Figure:**
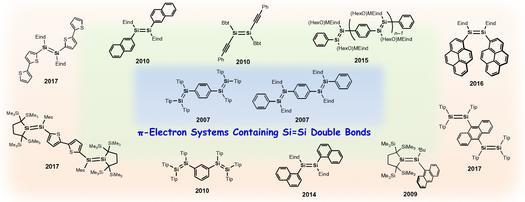

## 1. Introduction

In the periodic table, silicon is located just below carbon in the Group 14 elements, but carbon and silicon have different roles and functions in nature. While carbon is a central element in the organic substances that constitute the body of all living things, silicon is a key element of inorganic substances that constitute the earth’ s crust and is widely used in glass, semiconductors, concrete, ceramics, etc. Strangely, there is no organosilicon material containing C–Si bonds in nature except for silicon carbide in meteorites. Therefore, every organosilicon compound is an artificial substance created by human technology.

While a variety of allotropes of carbon, such as diamond, graphite, fullerenes, and carbon nanotubes, are known as a stable material mainly consisting of the *sp* ^3^- or *sp* ^2^-hybridized carbon atoms, the stable form of silicon only possesses a diamond-type structure based on the formally *sp* ^3^-hybridized silicon atoms. Can *sp* ^2^-hybridized silicon atoms exist as a stable substance? In recent years, silicene, the silicon analog of graphene, has attracted much attention, from both experimentalists and theoreticians, as a new two-dimensional allotrope of silicon [1–3]. Theoretical studies predict that repeating units in pure silicon nanosheets do not exhibit a planar hexagonal geometry like graphene but a non-classical propellane motif, presumably due to the instability associated with *sp* ^2^-hybridized silicon atoms [4].

**Figure 1.**
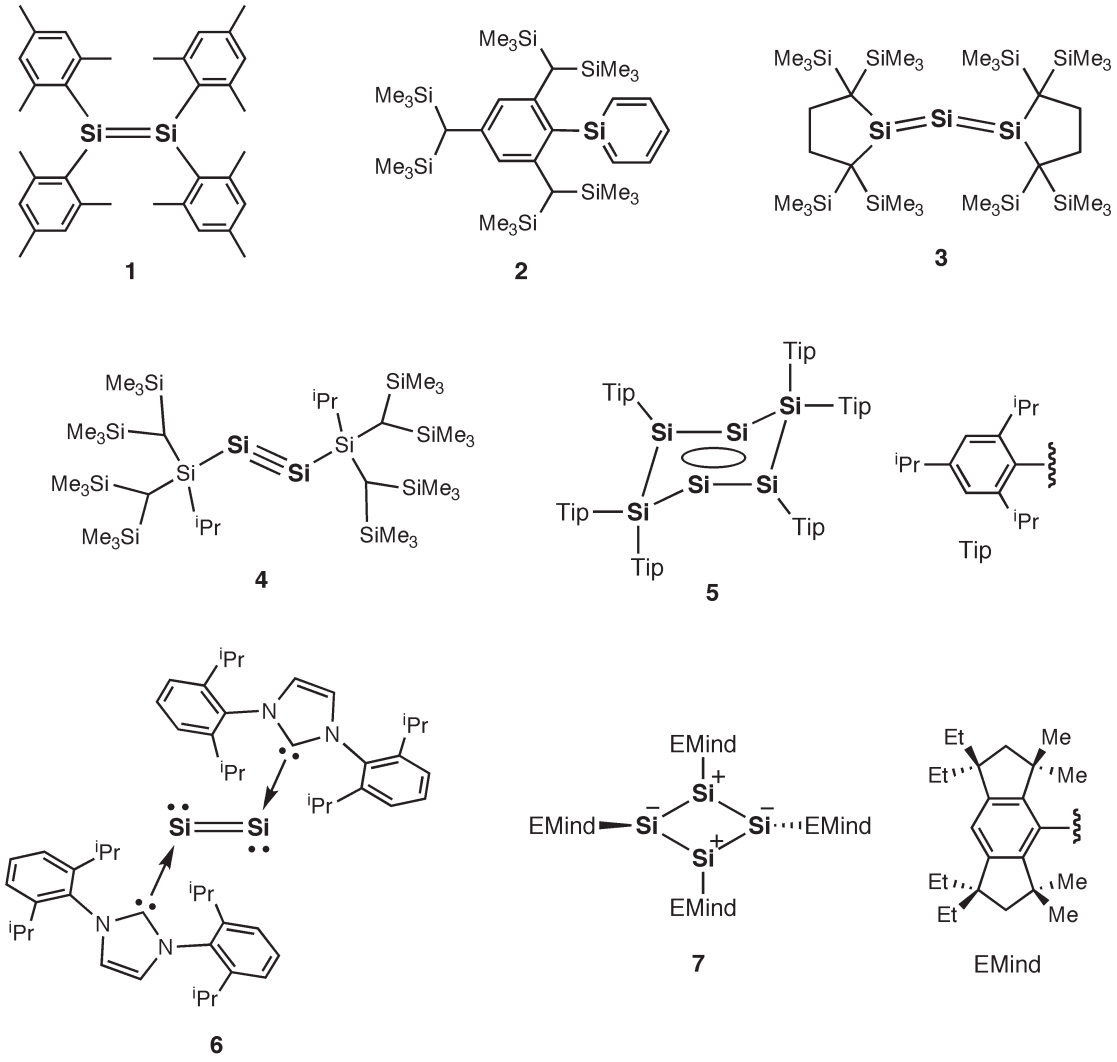
Examples of stable unsaturated silicon compounds.

In general, the Si–Si π-bond is much weaker than the C–C π-bond essentially due to the less effective overlap of the two adjacent 3*p* orbitals relative to that of the 2*p* orbitals, corresponding to the greater covalent atomic radius of silicon (1.11(2) Å) than that of carbon (*sp* ^3^-C; 0.76(1), *sp* ^2^-C; 0.73(2), and *sp*-C; 0.69(1) Å) [5]. In 1981, West, Fink, and Michl demonstrated for the first time that disilene (R_2_Si=SiR_2_) (**1**), the silicon analog of alkene, can be created based on the concept of kinetic stabilization using sterically demanding substituents, protecting the highly reactive Si=Si double bond, as shown in Figure 1, which produced a significant change in the main group chemistry [6–11]. In fact, after the finding of this isolatable disilene **1**, many kinds of unsaturated compounds of silicon have been successfully obtained by virtue of the steric effects of the bulky protecting groups [12–22]. For recent representative examples, since 2000, silaaromatics (**2**) [23–25], trisilaallenes (R_2_Si=Si=SiR_2_) (**3**) [26,27], and disilynes (RSi≡SiR) (**4**) [28–31] have been isolated using the appropriately designed bulky aryl, alkyl, and silyl substituents, respectively. Also, a tricyclic aromatic isomer of hexasilabenzene (**5**) [32] and a disilicon(0) fragment coordinated by the *N*-heterocyclic carbenes (NHCs) (**6**) [33] were synthesized as stable crystalline compounds. In 2011, we reported the synthesis of a cyclobutadiene (CBD) silicon analog, i.e. tetrasilacyclobutadiene (**7**), with a planar rhombic charge-separated structure originating from the polar Jahn–Teller distortion [34]. This is the first persila[*n*]annulene compound, (SiR)_*n*_ (*n* is an even number equal to or greater than 4), with a cyclic structure consisting of formally *sp* ^2^-hybridized silicon atoms, which will open a new facet of silicon π-science.

Although the fundamental chemistry of the low-coordinated compounds of silicon has been steadily established year-by-year, the functions and applications of the Si–Si π-bonds consisting of the 3*p*π electrons on the formally *sp* ^2^-hybridized silicon atoms have rarely been explored. In main group element chemistry, recent synthetic efforts have been directed toward investigating the combination of the multiple bonds of the heavier main group elements and the carbon π-electron systems due to their unique electronic properties and potentially useful technological applications for organic electronics, which would offer a new avenue to functional organoelement materials [35–45]. However, this chemistry always faces a formidable challenge. While the sufficient steric effects of the bulky substituents are crucial in protecting the highly reactive heavier multiple bonds, it may cause twisting of the π-framework, which prevents the preferred extension of the π-conjugation over the skeleton.

**Figure 2.**
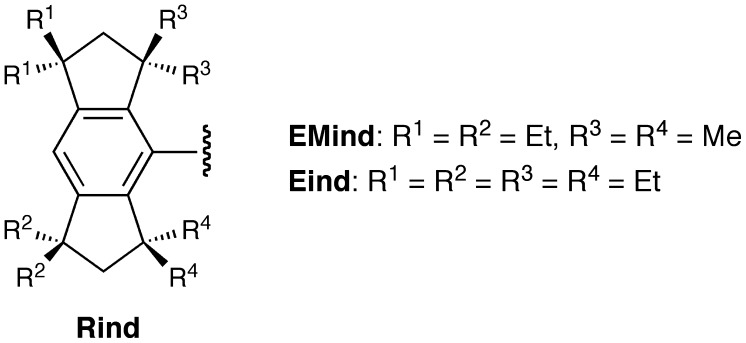
Rind groups.

In order to further develop this chemistry toward advanced materials science and technology, we have designed a series of fused-ring bulky 1,1,3,3,5,5,7,7-octa-R-substituted *s*-hydrindacen-4-yl groups, called the ‘Rind’ groups, as shown in Figure 2, where R denotes the initial of the substituents on the benzylic positions of the hydrindacene skeleton [46]. The Rind groups are actually giant aryl hydrocarbon substituents. Nevertheless, they can be easily prepared by organic synthetic methods, including the intramolecular Friedel-Crafts double cyclization [47]. In addition, various R groups (R^1^, R^2^, R^3^, and R^4^ groups) can be introduced at the four benzylic positions of the hydrindacene skeleton. While the peripheral R^1^ and R^2^ groups can control their physical properties such as crystallinity and solubility, the proximal R^3^ and R^4^ groups can directly change the steric (size and shape) effects of the Rind groups. Also, the Rind groups have a rigid structure based on the fused-ring system and show a high chemical stability due to the full substitution at all the benzylic positions, whose C–H bonds are generally more reactive than other C–H bonds. The term ‘Rind’ in English describes the thick outer skin of some types of fruits such as an orange and melon, which is fully in accordance with our research idea, i.e. *Rind can keep the inside fresh*. Actually, the Rind groups provide us great opportunities to study a variety of low-coordinate compounds of the main group elements [34,48–62] and coordinatively unsaturated transition metal complexes [63–71].

**Figure 3.**
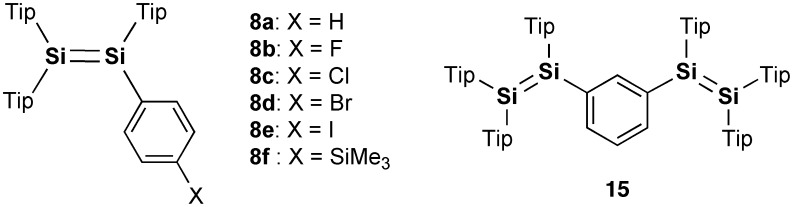
Disilenes **8** and **15**.

**Figure 4.**
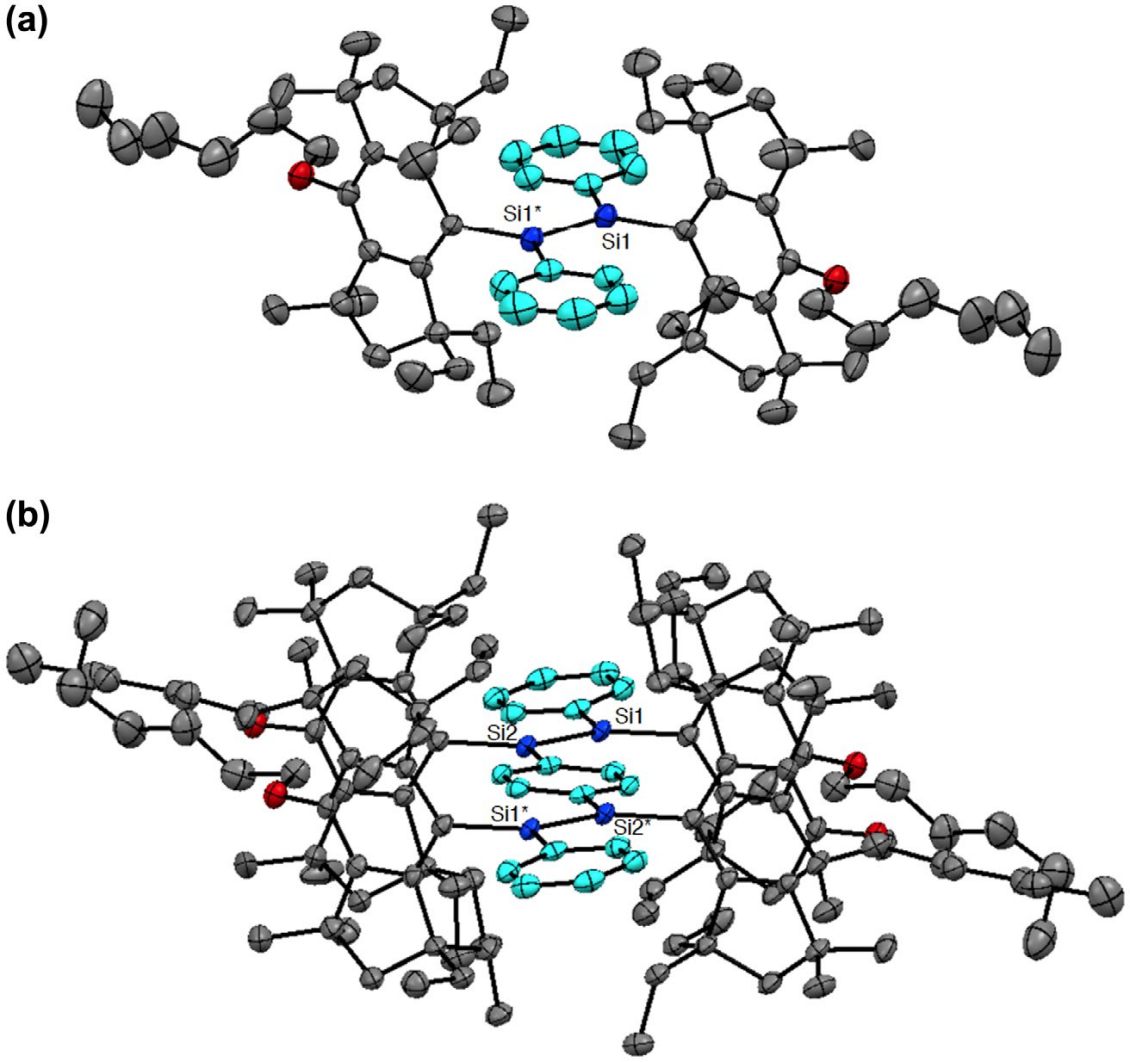
Molecular structures of **16** (a) and **17** (b) determined by X-ray crystallography.

**Figure 5.**
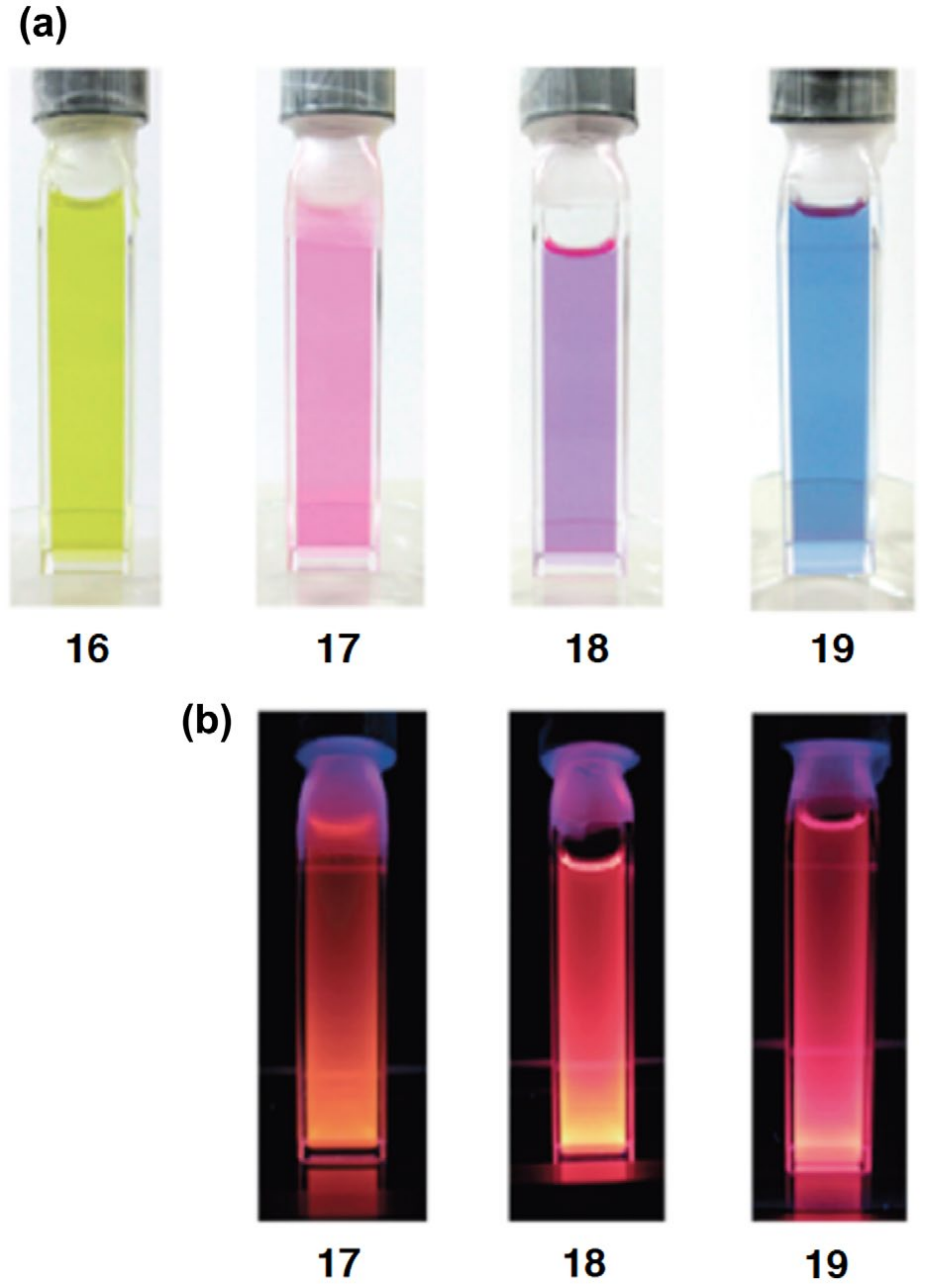
Photographs of the THF solutions of **16**–**19** at room temperature: (a) under room light; (b) under 360 nm UV light.

**Figure 6.**
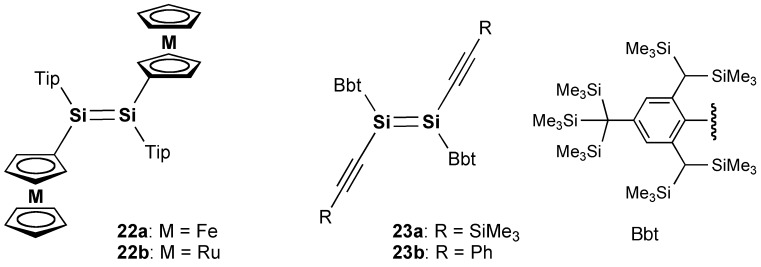
Disilenes **22** and **23**.

**Figure 7.**
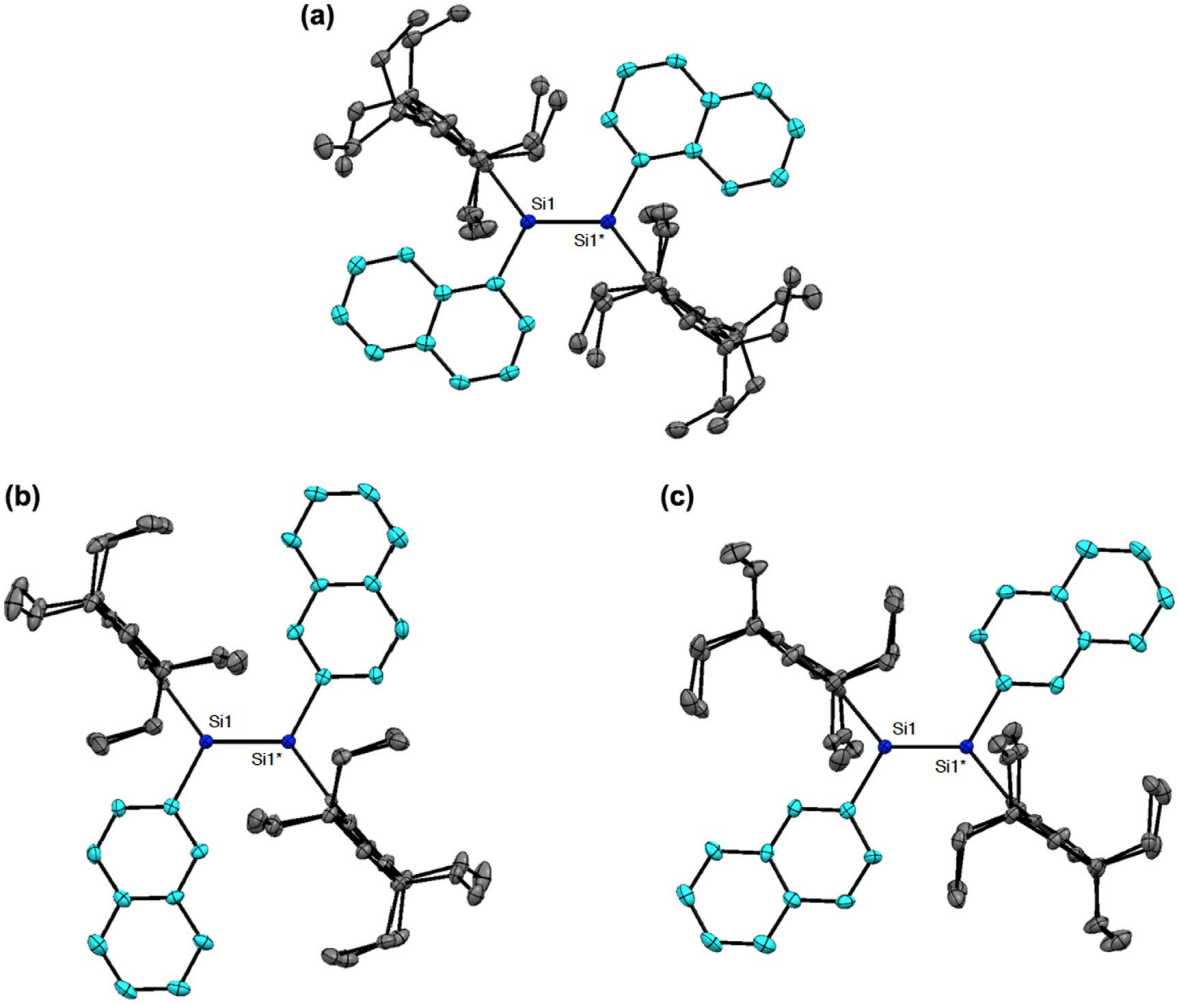
Molecular structures of **24** (a), **25a** (b), and **25b** (c) determined by X-ray crystallography.

**Figure 8.**
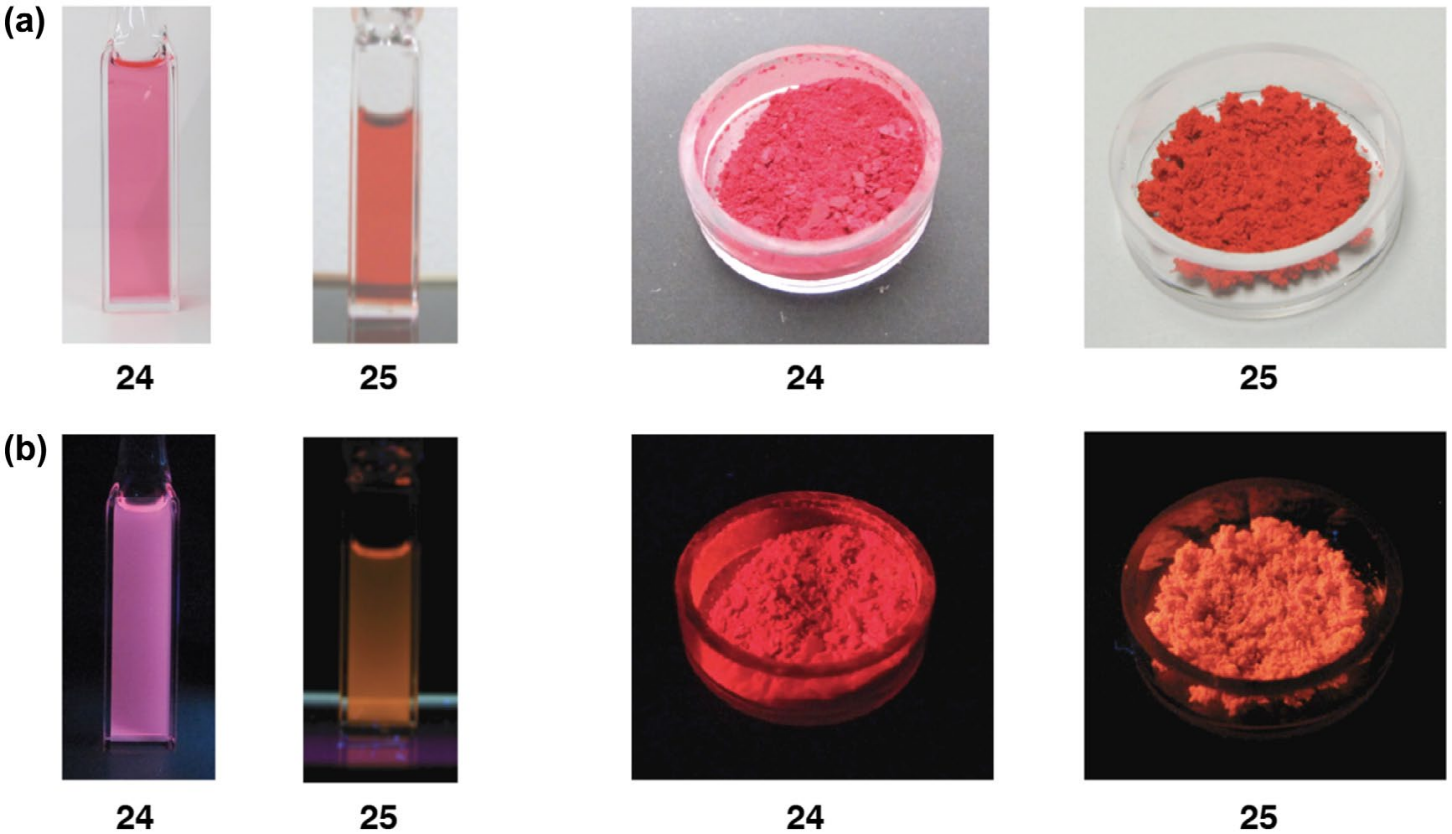
Photographs of the THF solutions (left) and solid in the air (right) of **24** and **25**: (a) under room light; (b) under 365 nm UV light.

**Figure 9.**
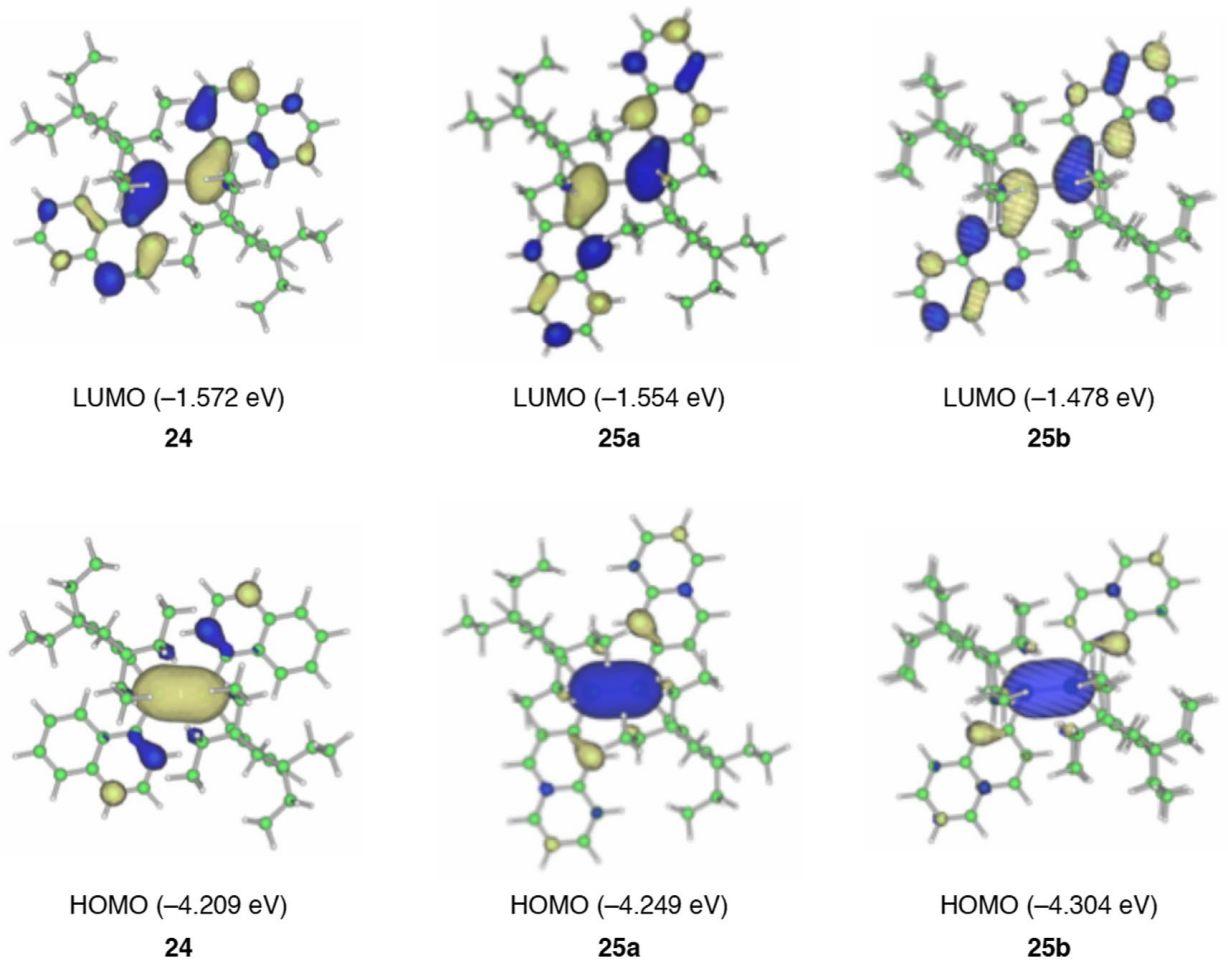
Frontier molecular orbitals of **24**, **25a**, and **25b** together with the energy levels.

**Figure 10.**
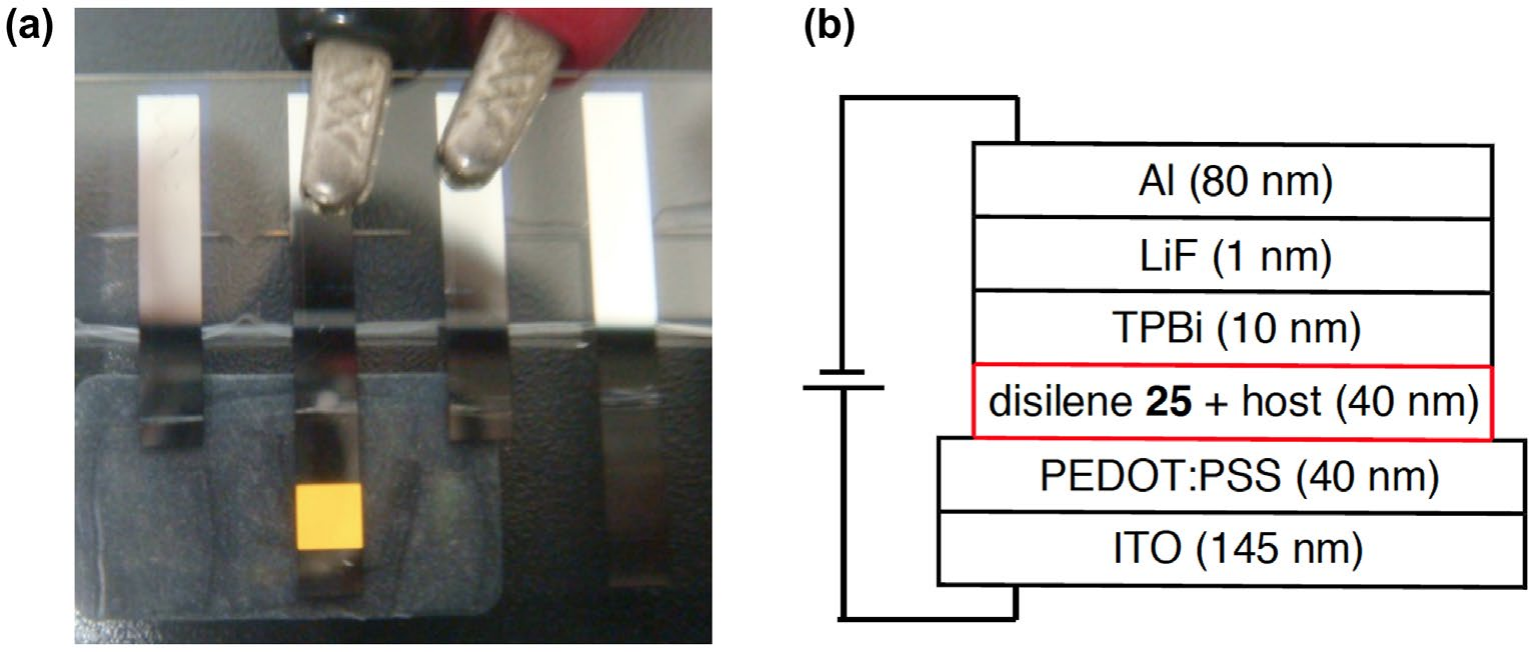
(a) The configuration of the EL device; (b) EL from the device at a 5 V applied voltage.

**Figure 11.**
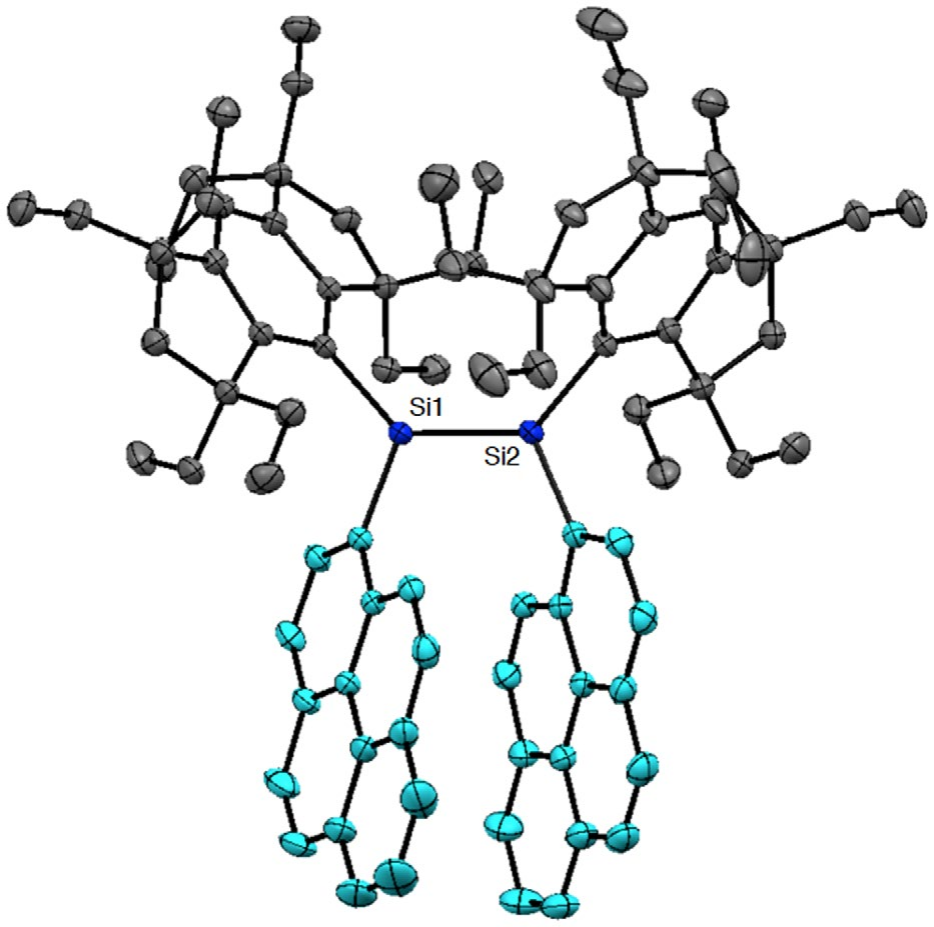
Molecular structure of **29** determined by X-ray crystallography.

**Figure 12.**
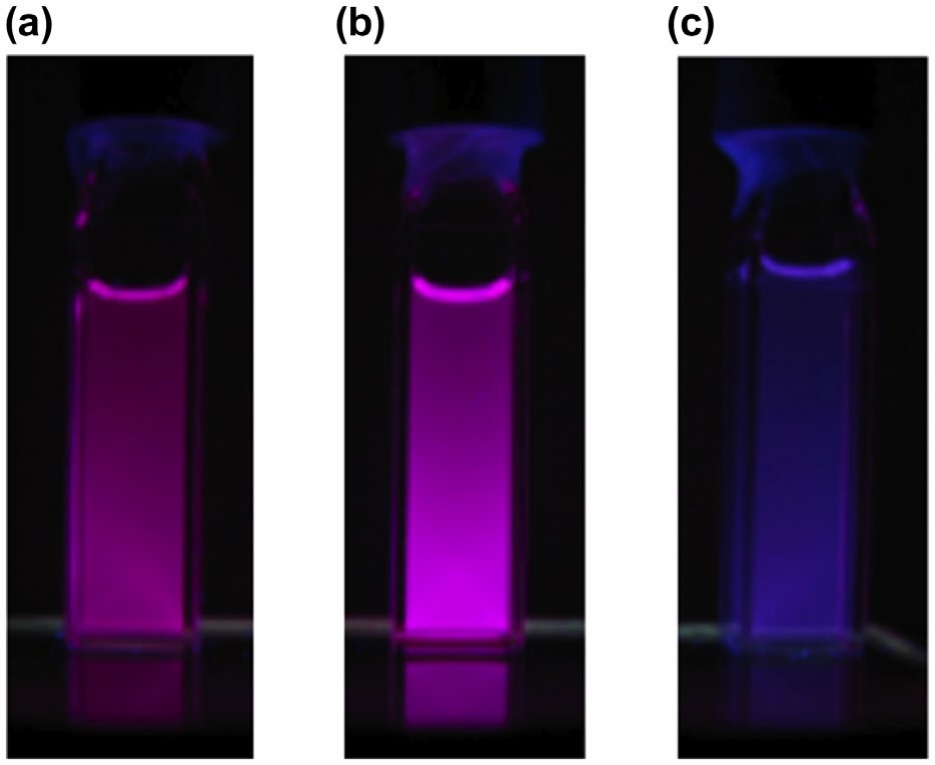
Photographs of the solutions of **29** under 365 nm UV light: (a) hexane; (b) THF; (c) acetone.

**Figure 13.**
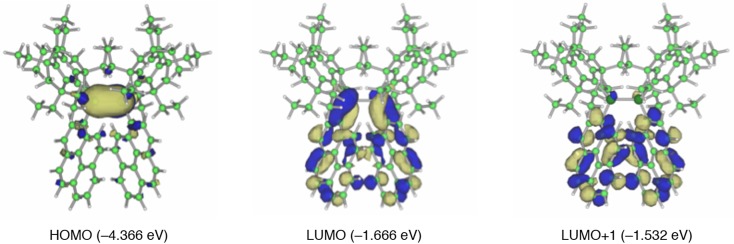
Selected molecular orbitals of **29** together with the energy levels.

**Figure 14.**
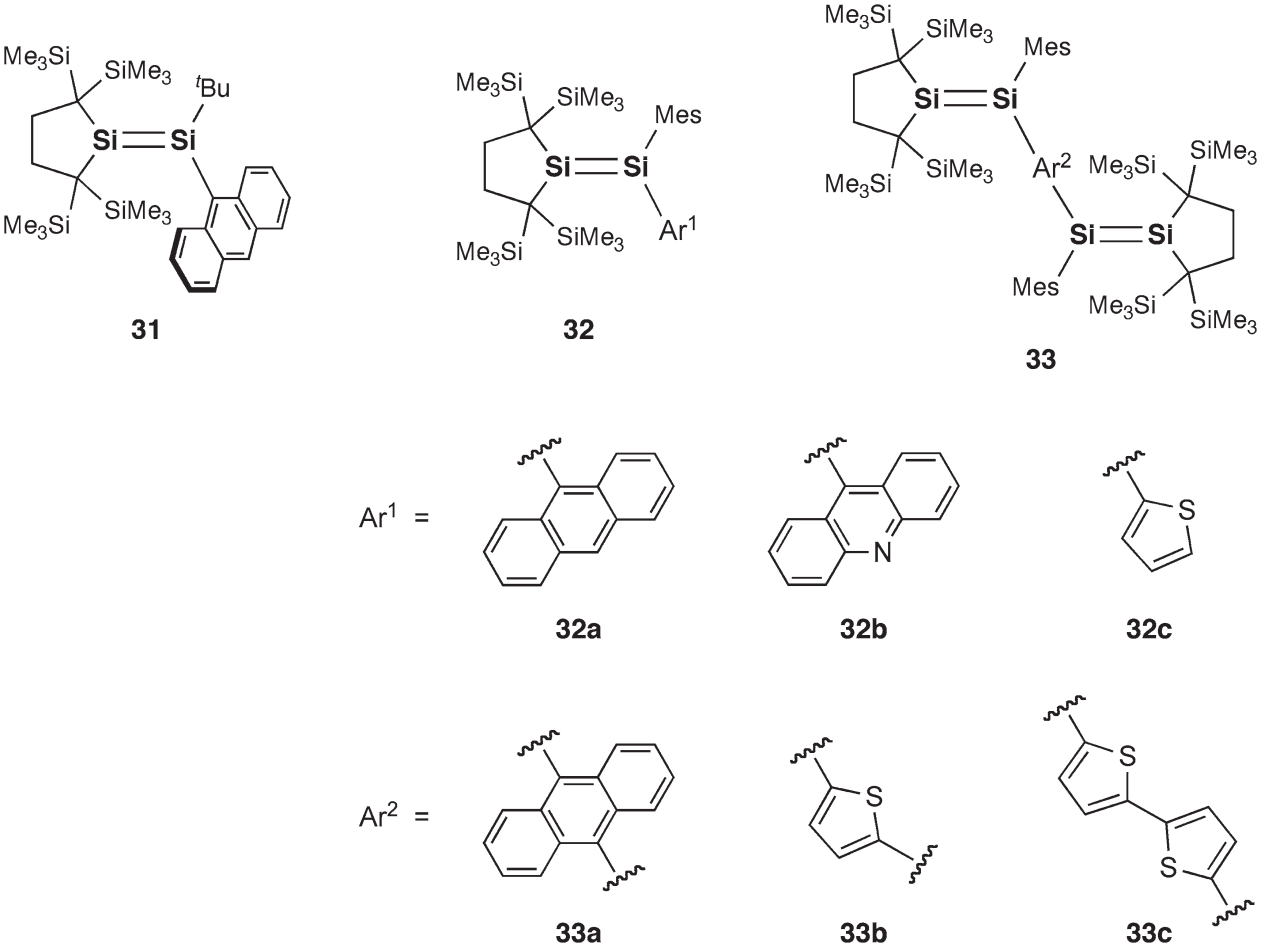
Disilenes **31**–**33**.

**Figure 15.**
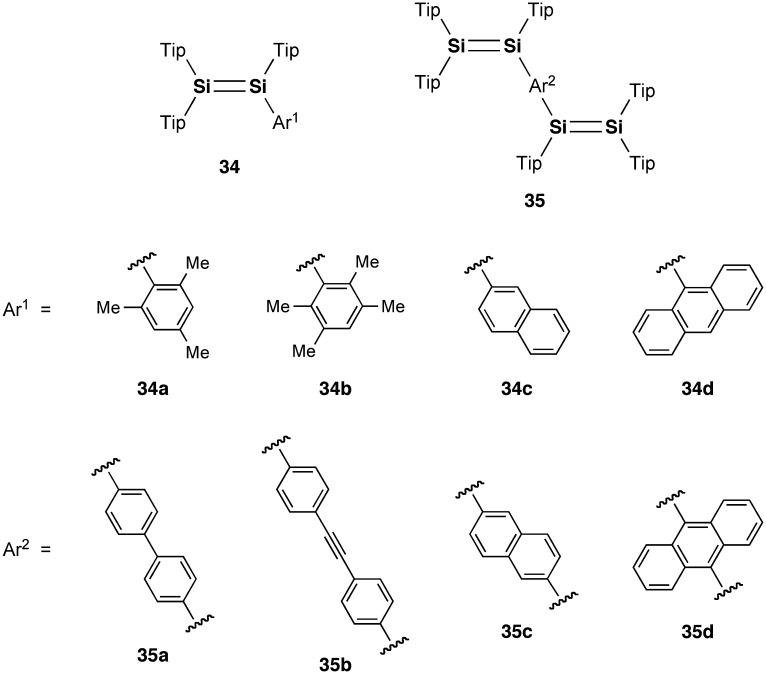
Disilenes **34** and **35**.

**Figure 16.**
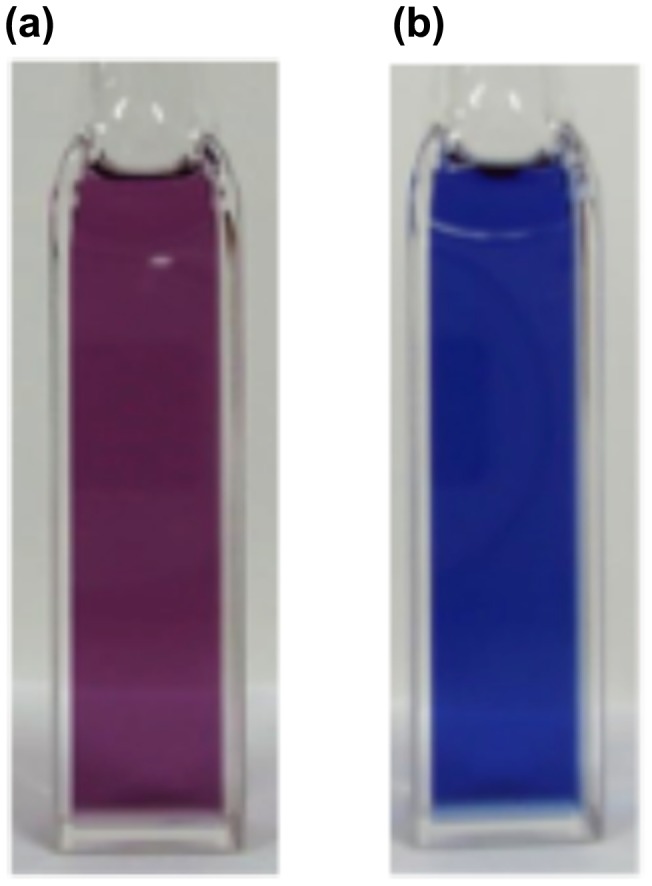
Photographs of the THF solutions of **29**: (a) before irradiation; (b) after irradiation at 530 nm.

**Figure 17.**
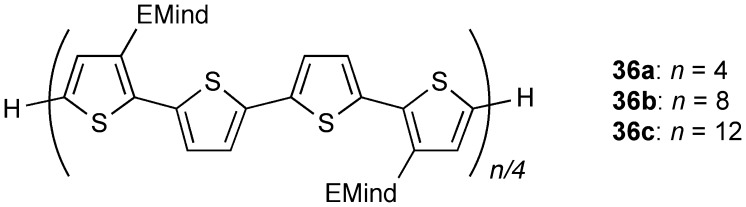
Oligothiophenes **36**.

**Figure 18.**
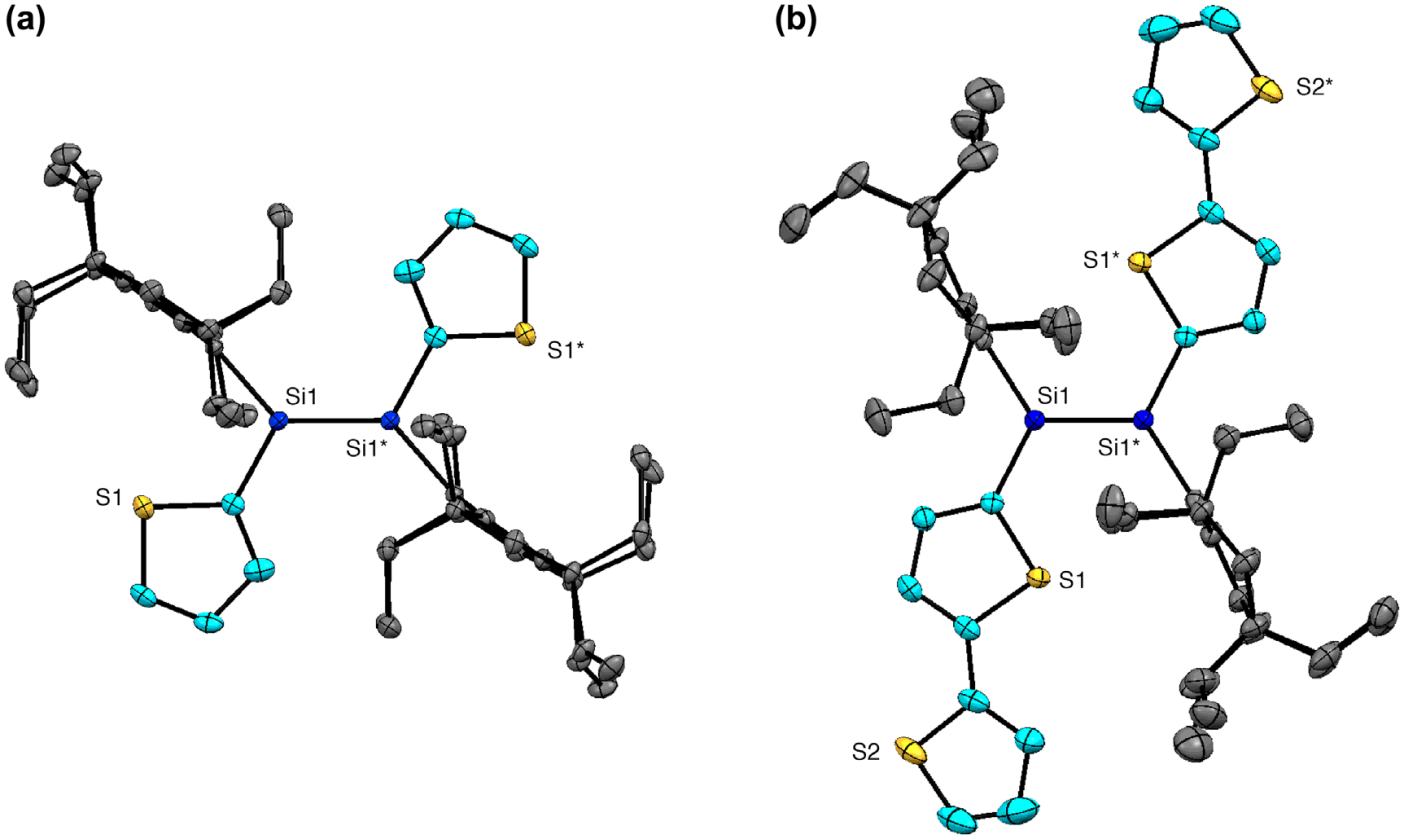
Molecular structures of (a) **37a** and (b) **38a** determined by X-ray crystallography.

**Figure 19.**
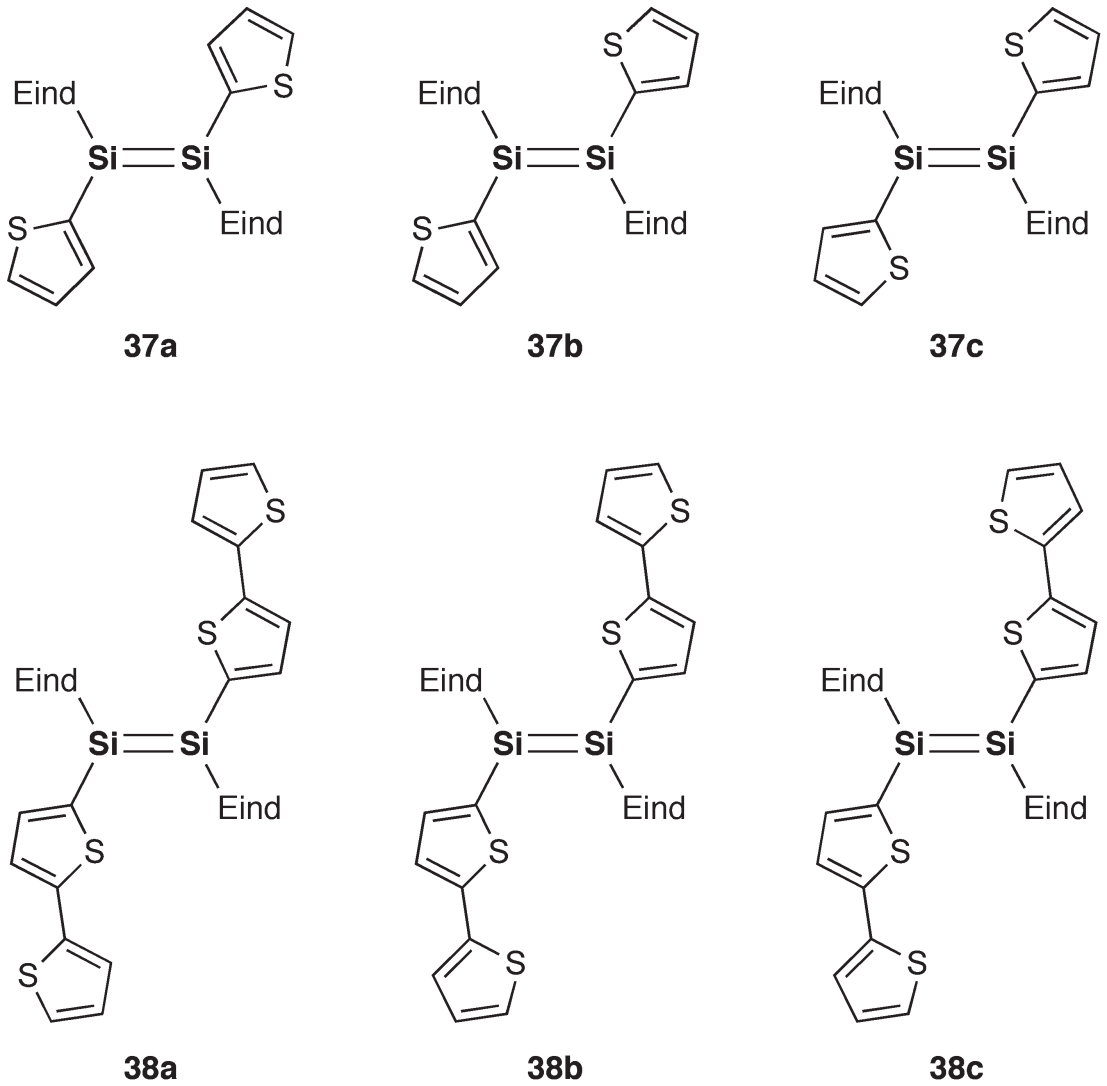
Rotational isomers of **37** and **38**.

**Figure 20.**
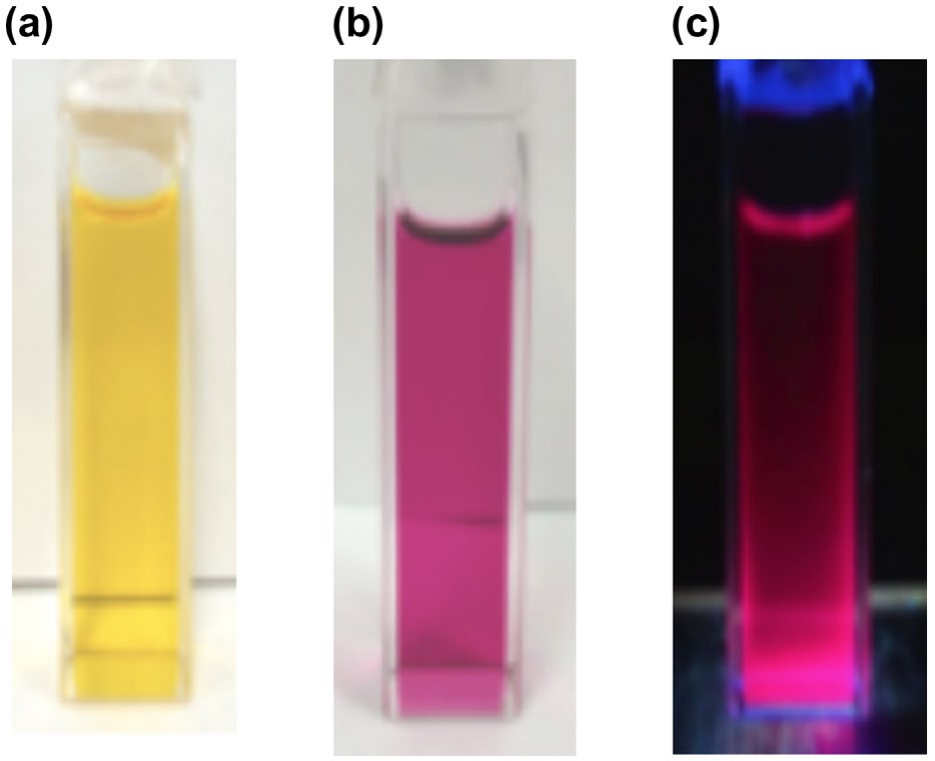
Photographs of the THF solutions: (a) **37** under room light; (b) **38** under room light; (c) **38** under 365 nm UV light.

**Figure 21.**
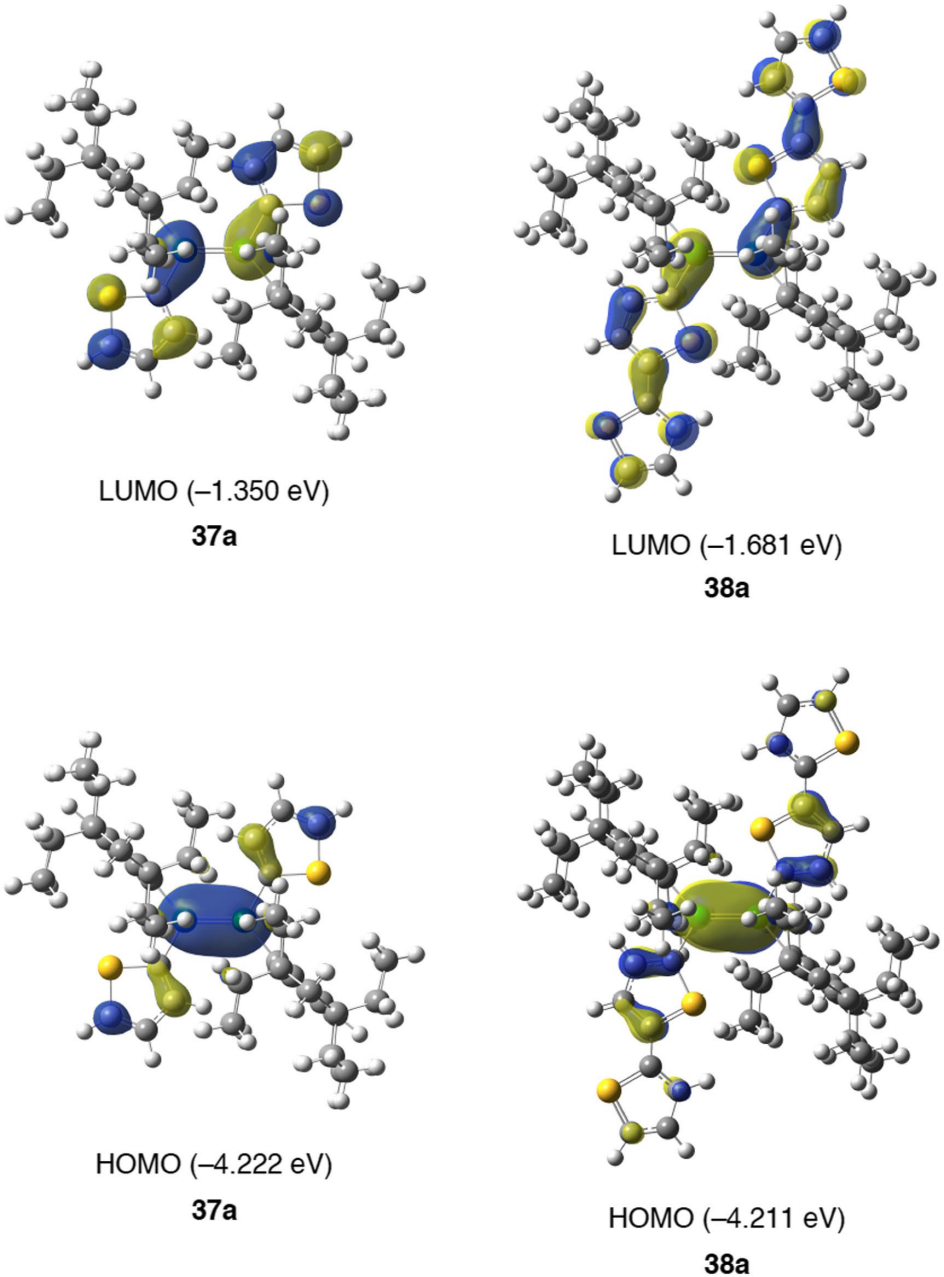
Frontier molecular orbitals of **37a** and **38a** together with the energy levels.

**Figure 22.**
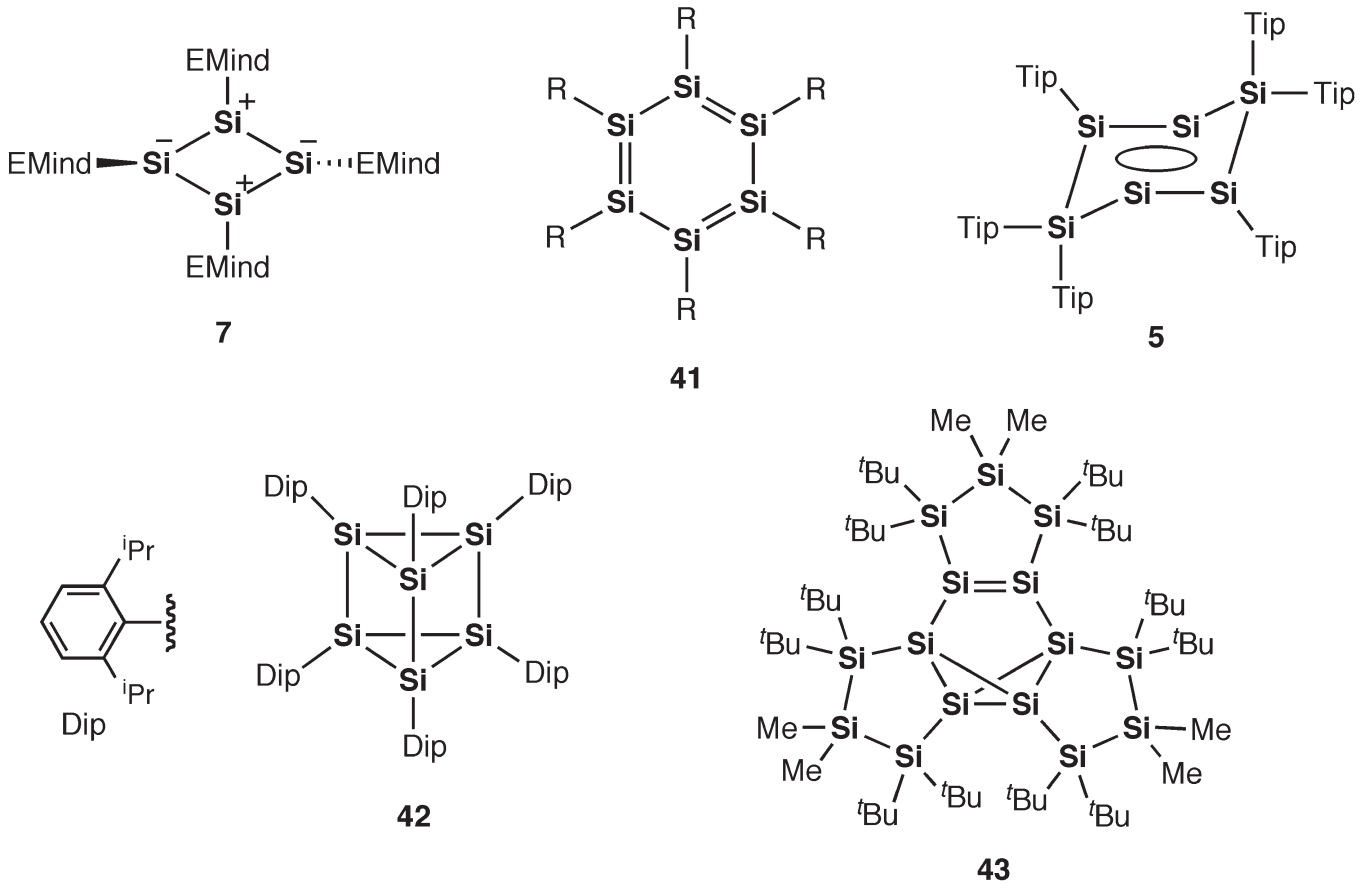
Compounds **5**, **7**, and **41**–**43**.

**Figure 23.**
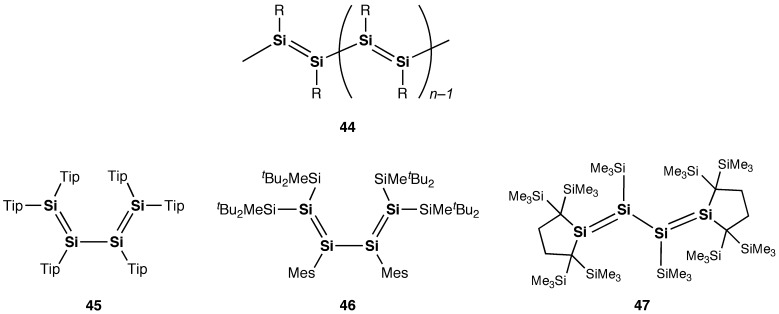
Compounds **44**–**47**.

In this review, we describe the recent progress in developing organic π-electron architectures featuring Si=Si double bonds, mainly focusing on the following four topics: (1) oligo(*p*-phenylenedisilenylene)s; π-conjugation between benzene rings and Si=Si units, (2) air-stable emissive disilenes with naphthyl groups; potential applicability in electroluminescence devices, (3) disilene π-system with pyrenyl groups; evidence for intramolecular charge-transfer emission, and (4) disilene–thiophene π-systems; future organosilicon chemistry for developing advanced materials.

## 2. Oligo(*p*-phenylenedisilenylene)s; π-conjugation between benzene rings and Si=Si units

Poly(*p*-phenylenevinylene)s (PPVs) with alternating benzene rings and C=C double bonds are some of the most attractive conducting polymers due to their excellent stability and processability and unique electronic and optical properties, which can be used for various applications in modern electrochemistry [72,73]. Oligo(*p*-phenylenevinylene)s (OPVs) have also received extensive attention as linear monodispersed π-conjugated oligomers with well-defined molecular structures and tunable optoelectronic properties [74–76]. Since the Si=Si units possess a narrower energy gap between the highest occupied molecular orbital and the lowest unoccupied molecular orbital (HOMO–LUMO) relative to the C=C units [12–22], the disilene analogs of OPVs, oligo(*p*-phenylenedisilenylene)s (Si–OPVs), would provide new opportunities for application to a range of organic electronic devices.

**Scheme 1.**
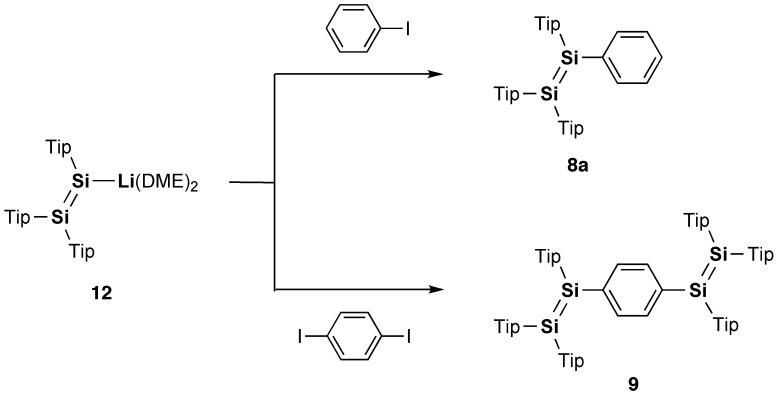
Synthesis of compounds **8a** and **9**.

**Scheme 2.**
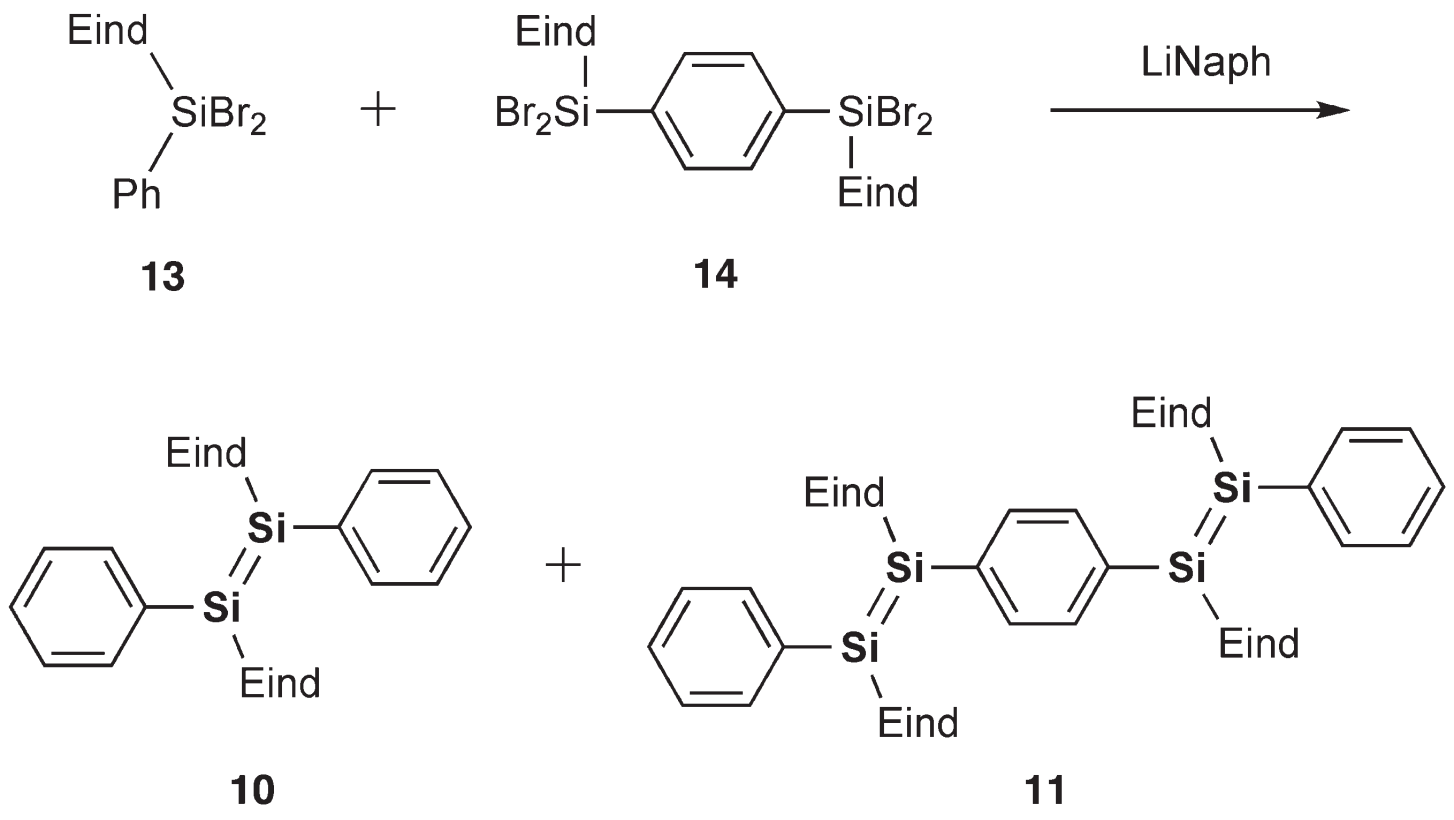
Synthesis of compounds **10** and **11**.

In 2007, model systems of the Si–OPVs (**8a** and **9**–**11**) were synthesized by the groups of Scheschkewitz and Tamao employing the bulky Tip (2,4,6-triisopropylphenyl) and Eind (R^1^ = R^2^ = R^3^ = R^4^ = Et) groups [77,78]. As shown in Scheme 1, the phenyl-substituted disilene, (Tip)_2_Si=Si(Tip)Ph (**8a**), and the *para*-phenylene-bridged tetrasiladiene **9** can be obtained as yellow and red crystals in 58% and 72% yields by the reaction of the isolatable disilenyllithium, (Tip)_2_Si=Si(Tip)Li(DME)_2_ (DME = 1,2-dimethoxyethane) (**12**) [79], which is a silicon analog of vinyllithium, with iodobenzene and 1,4-diiodobenzene. In contrast, compounds **10** and **11** have been prepared by the reductive co-condensation of the two kinds of dibromosilanes, (Eind)PhSiBr_2_ (**13**) and [(Eind)SiBr_2_]_2_(1,4-C_6_H_4_) (**14**), with a 5:1 molar ratio using lithium naphthalenide (LiNaph) as a homogeneous reducing agent (Scheme 2), in which **13** and **14** serve as the end-capping unit and central building unit for the Si–OPVs, respectively. The resulting monomer **10** and dimer **11** can be separated by silica gel column chromatography in a glove box using hexane and toluene as the eluents, leading to the isolation of yellow-orange crystals of **10** in 35% yield and red crystals of **11** in 15% yield. However, the higher oligomers, such as the trimer and tetramer, could not be obtained in a pure form mainly due to their poor solubility in common organic solvents.

In addition to the initial achievements, Scheschkewitz et al. prepared a series of *para*-functionalized-phenyl-substituted disilenes, (Tip)_2_Si=Si(Tip)(4–X–Ph) (X = F (**8b**), Cl (**8c**), Br (**8d**), I (**8e**), SiMe_3_ (**8f**)) [80], which are shown in Figure 3. The laterally functionalized disilenes **8b–e** can be synthesized by a similar reaction between the disilenyllithium **12** and the *para*-functionalized-phenyl iodides (4–X–PhI). In addition, the *para*-trimethylsilylphenyldisilene **8f** has been obtained as a major product by the reaction of the *para*-bromophenyldisilene **8d** with 2 equiv of *tert*-butyllithium (^*t*^BuLi) followed by the addition of trimethylchlorosilane (Me_3_SiCl). The disilenes **8a–d** exhibit a good correlation of the ultraviolet–visible (UV–vis) absorptions with the electronic Hammett parameters. As shown in Figure 3, the *meta*-phenylene-bridged tetrasiladiene (**15**), which is a regioisomer of **9**, has also been isolated as orange crystals in 85% yield by the reaction of **12** with 1,3-diiodobenzene [80].

**Scheme 3.**
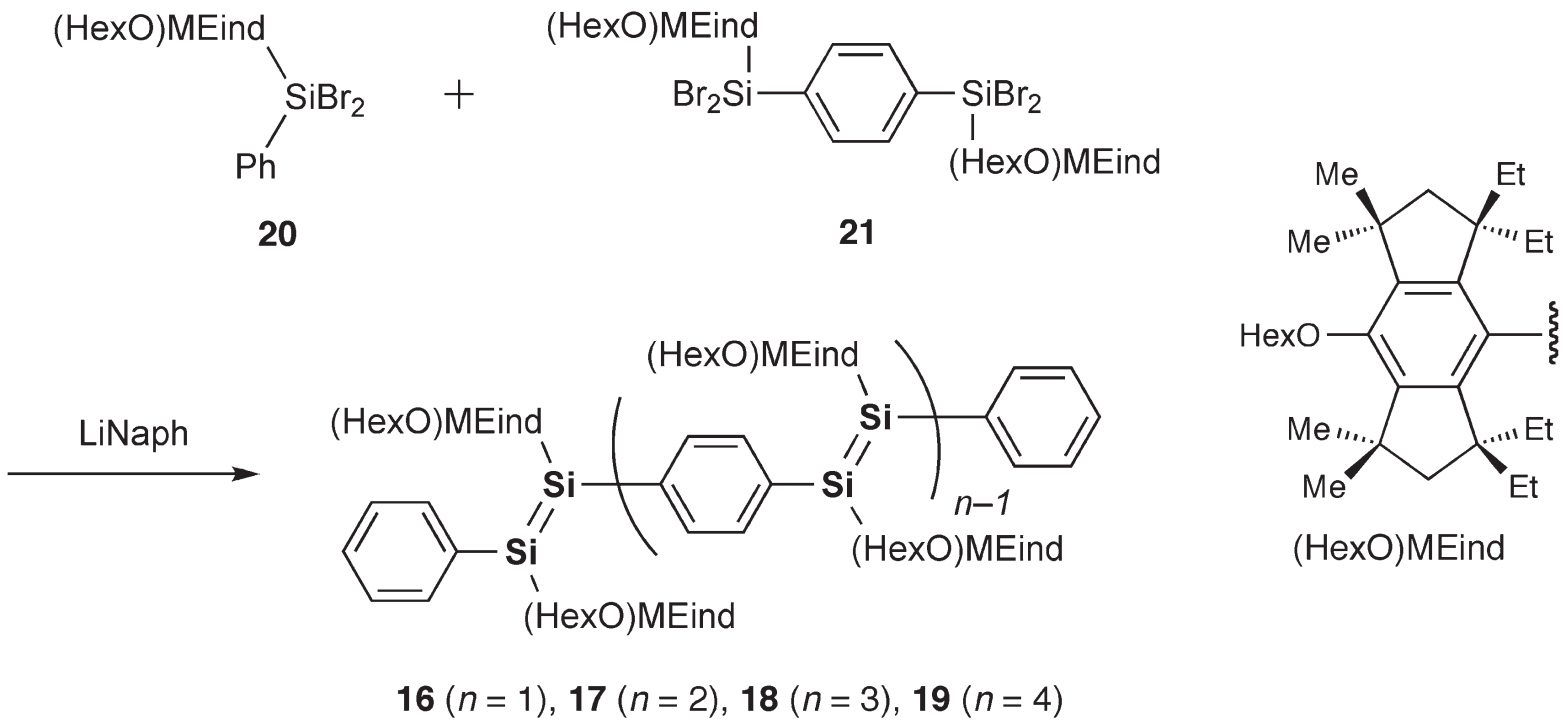
Synthesis of compounds **16**–**19**.

As shown in Scheme 3, we have recently obtained the new Si–OPVs (**16**–**19**) having the modified Rind group, (HexO)MEind group, with a hexyloxy chain at the *para* position of the MEind (R^1^ = R^2^ = Me, R^3^ = R^4^ = Et) group for improving their solubility [81]. A similar one-pot reductive co-condensation reaction of [(HexO)MEind]-PhSiBr_2_ (**20**) and [{(HexO)MEind}SiBr_2_]_2_(1,4-C_6_H_4_) (**21**) in a 2:1 molar ratio has led to the successful isolation of the yellow crystals of the monomer **16** in 10% yield, deep red crystals of the dimer **17** in 39%, a purple powder of the trimer **18** in 7% yield, and a deep purple powder of the tetramer **19** in 5% yield, which can be separated by silica gel column chromatography in a glove box using toluene and tetrahydrofuran (THF) as the eluents.

Figure 4 shows the molecular structures of **16** and **17** based on the single-crystal X-ray diffraction analysis. The bulky (HexO)MEind groups effectively encapsulate the reactive Si=Si units and produce the highly coplanar Si–OPVs π-frameworks. The selected structural parameters of **16** and **17** are summarized in Table 1, together with those of **8a**–**d**, **9**–**11**, and **15** for comparison. The disilene cores of **16** and **17** display an almost planar geometry. The *trans*-bent angles (*θ*) between the Si–Si vector and the C–Si–C plane are estimated to be 1.65(8)° for **16** and 0.62(12) and 3.38(13)° for **17**, which are comparable to those of **10** (*θ* = 2.72(14)°) and **11** (*θ* = 0.7(3) and 2.7(3)°) and much smaller than those of **8a** (*θ* = 22.8 and 22.0°), **9** (*θ* = 16.45(10) and 19.31(10)°), and **15** (*θ* = 20.3 and 26.7°). These X-ray data show the excellent structural controllability of the Rind groups, where the proximate ethyl side chains on the *s*-hydrindacene skeletons can interlock with one another above and below the Si=Si moieties to enforce the planar structure.

**Table 1.** X-ray structural parameters of disilenes.

Compound	*Trans*-bent angle (*θ*) (deg)	Si=Si bond length (Å)	References
**1**	12, 14	2.143(2)	10, 11, 19
**1**•C_7_H_8_	18	2.160(1)	7–9, 11, 19
**1**•THF	0	2.146	11, 19
**8a**	22.8, 22.0	2.175(1)	77
**8b**	5.5, 5.9	2.147(1)	80
**8c**	15.8, 23.8	2.1735(4)	80
**8d**	16.2, 24.5	2.1707(5)	80
**9**	16.45(10), 19.31(10)	2.1674(8)	77
**10**	2.72(14)	2.1593(16)	80
**11**	0.7(3), 2.7(3)	2.156(2)	80
**15**	20.3, 26.7	2.189(1)	80
**16**	1.65(8)	2.1626(8)	81
**17**	0.62(12), 3.38(13)	2.1642(8)	81
**22a**	27.9	2.1735(15)	87
**22b**	32.3	2.1851(12)	88
**23a**	8.9	2.202(2)	89
**23b**	11.3	2.1871(10)	89
**24**	4.93(12)	2.1688(7)	90
**25a**	2.25(14)	2.1623(18)	91
**25b**	9.57(11)	2.1667(12)	91
**29**	8.22(8), 2.96(8)	2.1718(6)	97
**31**	11.4, 6.9	2.1754(12)	100
**32a**	4.0, 4.9	2.1697(6)	101
**32b**	7.5, 7.7	2.1669(5)	101
**32c**	10.5, 26.2	2.1826(6)	101
**33b**	15.5, 16.1, 26.0, 26.0	2.1846(5), 2.1829(5)	101
**33c**	11.9, 20.2	2.176(4)	101
**34a**	6.7, 7.2	2.1453(6)	102
**34b**	7.5, 1.7	2.1516(7)	102
**34c**	11.6, 3.2	2.1525(6)	102
**35a**	6.7, 4.2	2.1460(8)	102
**35b**	15.3, 9.7	2.1530(8)	102
**35c**	5.5, 1.3	2.1622(6)	102
**35d**	4.7, 3.5	2.1483(8)	102
**37**	19.12(12), 13.5(6)	2.1712(11)	86
**38**	5.44(10)	2.1584(9)	86

The photophysical data of the Si–OPVs (**16**–**19**) are summarized in Table 2. The absorption color gradually changes from yellow for **16** to blue for **19**, as shown in Figure 5(a). The UV–vis spectra in THF exhibit absorption maxima (*λ*_max_(abs)) at 465 nm for **16**, 546 nm for **17**, 581 nm for **18**, and 610 nm for **19**, some of which are comparable to those of the Eind-based Si–OPVs (461 nm for **10** and 543 nm for **11** in hexane). The high molar extinction coefficients of **16**–**19** (*ε* = 2.8–7.1 × 10^4^ cm^–1^ M^–1^) are assignable to the allowed HOMO → LUMO (π → π*) transitions, which are supported by the theoretical calculations using time-dependent density functional theory (TD-DFT) [82]. It should be noted that the *λ*_max_(abs) values of **10** (461 nm) and **16** (465 nm) are more than 40 nm red-shifted from that of tetramesityldisilene **1** (420 nm) [6], indicating the π-conjugation over the 1,2-diphenyldisilene (disilastilbene) skeleton. In addition, the considerable bathochromic shifts with the increasing Si=Si units are most likely interpreted in terms of the extended π-conjugation over the entire Si–OPVs skeleton, thus providing clear evidence for the efficient π-conjugation between the benzene rings and Si=Si double bonds. The *λ*_max_(abs) values provide a good fit to Meier’s equation [83], which enables the estimation of an effective conjugation length (ECL) of nine repeat units (*n*_ECL_ = 9) and the absorption maximum of 635 nm for the infinite chain (*λ*_∞_(abs) = 635 nm). These estimated values (*n*_ECL_ = 9 and *λ*_∞_(abs) = 635 nm) for the Si–OPVs are lower and longer than those of the carbon counterpart, OPVs (*n*_ECL_ = 11 and *λ*_∞_(abs) = 481 nm), respectively [84]. Thus, the inclusion of the Si=Si double bonds into the carbon π-conjugated systems may cause a significant change in the electronic and absorption properties.

**Table 2.** Photophysical data for disilenes.

Compound	*λ*_max_(abs) (nm) [*ε* (cm^–1^ M^–1^)]	*λ*_max_(ex) (nm)	Δ*ν*_Stokes_ (cm^–1^)	Φ_F_	References
**1** (hexane)	420 [1.0 × 10^4^]	505	4000	<0.01	6, 9
**8a** (hexane)	439 [1.9 × 10^4^]				77
**8b** (hexane)	437 [1.7 × 10^4^]				80
**8c** (hexane)	445 [1.9 × 10^4^]				80
**8d** (hexane)	447 [1.6 × 10^4^]				80
**9** (hexane)	508 [2.7 × 10^4^]				77
**10** (hexane)	461 [2.4 × 10^4^]				78
**11** (hexane)	543 [3.0 × 10^4^]	612	2080	0.10	78
**15** (hexane)	450 [3.9 × 10^4^]				80
**16** (THF)	465 [2.8 × 10^4^]				81
**17** (THF)	546 [4.3 × 10^4^]	613	2000	0.11	81
**18** (THF)	581 [5.0 × 10^4^]	643	1660	0.46	81
**19** (THF)	610 [7.1 × 10^4^]	668	1420	0.48	81
**22a** (hexane)	427 [2.4 × 10^4^]				87
**22b** (hexane)	430 [2.2 × 10^4^]				88
**23a** (hexane)	437 [2.4 × 10^4^]				89
**23b** (hexane)	469 [3.1 × 10^4^]				89
**24** (THF)	521 [9.5 × 10^3^]	614	2910	<0.01	90
**24** (solid)		635		0.05	90
**25** (THF)	504 [2.5 × 10^4^]	586	2780	<0.01	91
**25** (solid)		619		0.23	91
**28** (THF)	590 [1.3 × 10^4^]				102
**29** (hexane)	506 [2.6 × 10^3^]	661		0.03	98
	566(sh) [2.0 × 10^3^]				
**29** (THF)	519(sh) [6.1 × 10^3^]	676		0.03	98
	575 [7.2 × 10^3^]				
**29** (acetone)	508 [4.6 × 10^3^]	694		0.03	98
	564(sh) [3.7 × 10^3^]				
**29** (solid)		712		0.04	98
**31** (3-MP^a^)	525 [420]				100
**31** (1,2-DCB^b^)	535 [480]				100
**32a** (hexane)	538 [1.4 × 10^3^]				101
**32b** (hexane)	583 [1.6 × 10^3^]				101
**32c** (hexane)	394(sh) [5.6 × 10^3^]				101
**33a** (hexane)	581 [2.55 × 10^3^]				101
**33b** (hexane)	439(sh) [4.0 × 10^3^]				101
**33c** (hexane)	441(sh) [7.0 × 10^3^]				101
**34a** (hexane)	430 [2.1 × 10^4^]				102
**34b** (hexane)	430 [1.7 × 10^4^]				102
**34c** (hexane)	463 [6.8 × 10^3^]				102
**34d** (hexane)	550 [3.8 × 10^3^]				102
**35a** (hexane)	463 [2.1 × 10^4^]				102
**35b** (hexane)	488 [4.6 × 10^4^]	570		<0.01	102
**35b** (solid)		619		0.015	102
**35c** (hexane)	484 [1.2 × 10^4^]	574		<0.01	102
**35c** (solid)		587		0.04	102
**35d** (hexane)	597 [7.5 × 10^3^]				102
**35d** (solid)		816		0.05	102
**37** (THF)	459 [1.1 × 10^4^]				86
**38** (THF)	530 [1.3 × 10^4^]	688	4330	0.01	86
**38** (solid)		691		0.11	86

^a^ 3-methylpentane.

^b^ 1,2-dichlorobenzene.

While the monomer **16** does not show any emission like **10**, the dimer **17**, trimer **18**, and tetramer **19** exhibit an intense fluorescence in THF at room temperature (Figure 5(b)). The emission maxima (*λ*_max_(ex)) are observed at 613 nm for **17**, 643 nm for **18**, and 668 nm for **19**, one of which is similar to that of **11** (612 nm). The quantum yields (Φ_F_) increased from 0.11 to 0.48 with the increasing number of repeated units. In contrast, the Stokes shifts (Δ*ν*_Stokes_) decreased from 2000 cm^–1^ for **17** to 1420 cm^–1^ for **19**, which are lower than those of the flexible carbon-based OPVs (3199–3029 cm^–1^) [83] and higher than those of the rigid carbon-bridged OPVs (772–583 cm^–1^) [85], suggesting the moderate rigidity of the Si–OPVs frameworks supported by the perpendicularly-fixed (HexO)MEind groups.

These studies reveal the possibility of constructing the π-conjugated disilene systems alternating the carbon-based 2*p*π and silicon-based 3*p*π-electrons, where the Si=Si double bonds would be promising building blocks. We hope that the present studies would provide a further challenge for the pure and applied chemistry of disilene copolymers containing various carbon π-electron systems. Actually, we very recently succeeded in obtaining some disilene–thiophene π-systems as model compounds for the disilene–thiophene copolymers [86], which are described in the latter part of this review.

## 3. Air-stable emissive disilenes with naphthyl groups; potential applicability in electroluminescence devices

After the initial findings of the model compounds of the Si–OPVs, the π-electron systems containing a Si=Si chromophore attracted much attention from the viewpoint of their potentially useful properties and unique functions. For example, as shown in Figure 6, a new type of disilene bearing metallocenyl groups has been synthesized by Sasamori, Tokitoh, and co-workers [87,88]. The 1,2-bis(metallocenyl)disilenes (**22**) exhibit a multistep redox process in the cyclic voltammograms, thus indicating the potential application of the disilene π-system as electrochemical materials. Tokitoh et al. have also shown that the 1,2-dialkynyldisilenes (**23**) can be obtained as a stable crystalline compound by taking advantage of the steric protection using the bulky Bbt groups (Bbt = 2,6-bis[bis(trimethylsilyl)methyl]-4-[tris(trimethylsilyl)methyl]phenyl) (Figure 6) [89]. The UV–vis spectrum of **23b** in hexane exhibits an absorption maximum (*λ*_max_(abs)) at 469 nm, which is 32 nm red-shifted compared with that of **23a** (437 nm), indicating the effective π-conjugation between the central Si=Si unit and two terminal phenyl groups via the C≡C triple bonds.

**Scheme 4.**
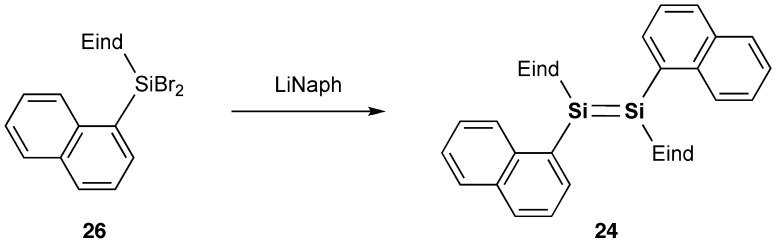
Synthesis of compound **24**.

**Scheme 5.**
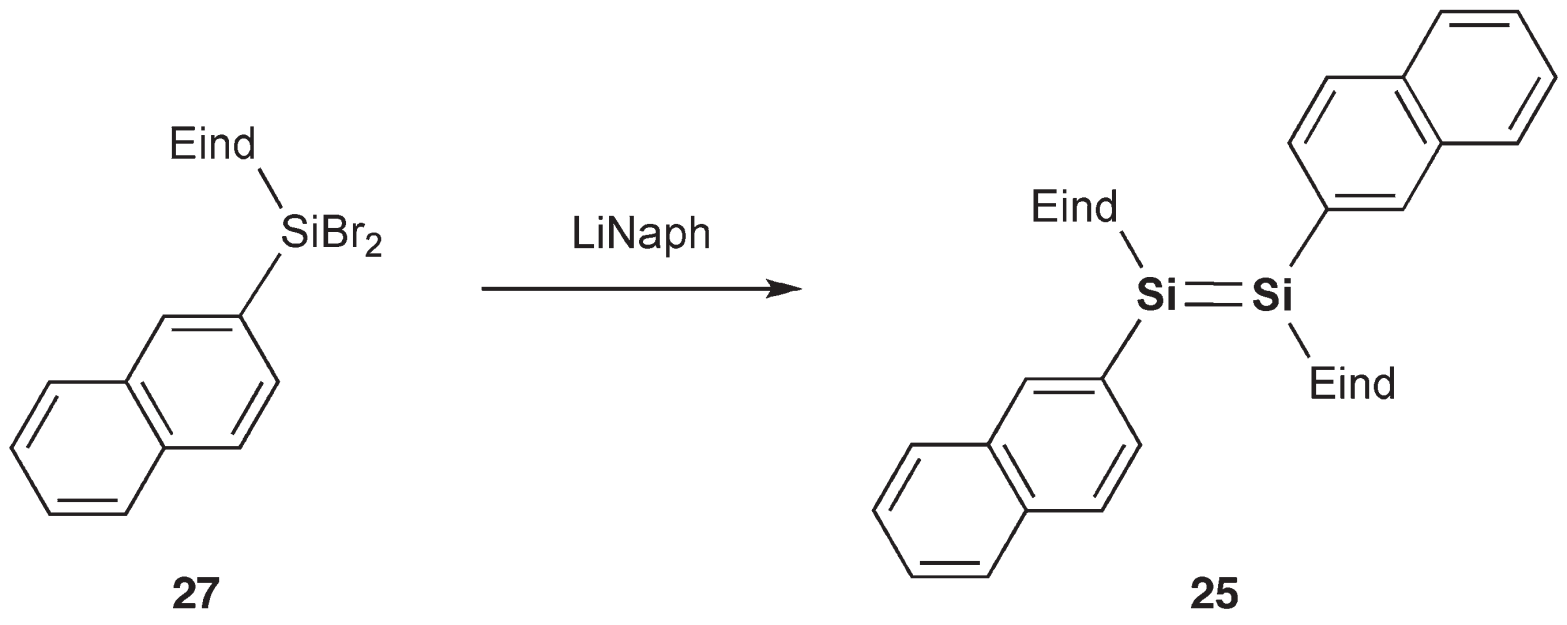
Synthesis of compound **25**.

We have set out to investigate the other types of disilene π-systems with two polycyclic aromatic groups on the Si=Si core incorporating the two bulky Rind groups. As the first target molecules, we designed two kinds of 1,2-dinaphthyldisilene regioisomers, (*E*)-1,2-di(1-naphthyl)disilene (**24**) and (*E*)-1,2-di(2-naphthyl)-disilene (**25**), as shown in Schemes 4 and 5 [90,91]. The disilenes **24** and **25** were obtained as red crystals in 59% and 57% yields, respectively, by the reductive coupling of (Eind)(1-naphthyl)SiBr_2_ (**26**) and (Eind)(2-naphthyl)SiBr_2_ (**27**). The disilenes **24** and **25** are extraordinarily air-stable in the solid state of more than several years with no detectable change as confirmed by the proton nuclear magnetic resonance (^1^H NMR) spectra, which indicates the excellent protection abilities of the Eind group. The disilenes **24** and **25** decompose in a dilute solution (*ca*. 10^–5^ mol L^–1^) upon exposure to air with a half-lifetime of 2–4 h, which is rather longer than that previously reported for (Tip)_2_Si=Si(Tip)_2_ (17 min) [92], as monitored by the UV–vis absorption spectroscopy.

Figure 7 shows the molecular structures of **24** and **25** confirmed by X-ray crystallography. Both molecules have an inversion center at the midpoint of the Si=Si double bond with an *E*-configuration. In the crystal of **24**, the hydrogen atoms at the *peri*-position on the 1-naphthyl groups participate in the CH–π interaction with the benzene ring of the perpendicularly-oriented Eind groups, producing the highly coplanar di(1-naphthyl)disilene skeleton, favorable for the efficient π-conjugation involving the Si=Si unit. In the crystal of **25**, the Si atoms and the 2-naphthyl groups are disordered over the two positions, which corresponds to a mixture of two rotational isomers, *s*-*cis*, *s*-*cis* (**25a**) and *s*-*trans*, *s*-*trans* (**25b**), with the occupancy factors of *ca*. 0.40/0.60. Each rotational isomer has an essentially coplanar di(2-naphthyl)disilene framework. The selected structural parameters of **24**, **25a**, and **25b** are summarized in Table 1. The *trans*-bent angles (*θ*) are estimated to be 4.93(12)° for **24**, 2.25(14)° for **25a**, and 9.57(11)° for **25b**. The Si=Si bond distance is 2.1688(7) Å for **24**, 2.1623(18) Å for **25a**, and 2.1667(12) Å for **25b**, which are comparable to those of **10** (2.1593(16) Å), **11** (2.156(2) Å), **16** (2.1626(8) Å), and **17** (2.1642(8) Å) and in the standard range of those reported for disilenes [15].

As shown in Figure 8, the disilenes **24** and **25** exhibit a strong absorption and emission at room temperature both in solution and in the solid state mainly due to the highly coplanar 1,2-dinaphthyldisilene π-frameworks. The photophysical data of **24** and **25** are summarized in Table 2. In the UV–vis spectra in THF, the absorption maxima (*λ*_max_(abs)) appear at 521 nm for **24** and 504 nm for **25**, which are red-shifted from that of **10** (461 nm), indicative of the effective π-conjugation over the dinaphthyldisilene skeletons. The emission maxima (*λ*_max_(ex)) are found at 614 nm for **24** and at 586 nm for **25**. The Stokes shift (Δ*ν*_Stokes_) values are estimated to be 2910 cm^–1^ for **24** and 2780 cm^–1^ for **25**, which are higher than that of **11** (2080 cm^–1^) but much lower than those of the tetramesityldisilene **1** (4000 cm^–1^) [8] and tetraneopentyldisilene (7300 cm^–1^) [93], thus indicating the structural rigidity of the dinaphthyldisilene skeletons. Each of the disilenes **24** and **25** shows a weaker emission in solution relative to that in the solid state mainly ascribed to the free-rotation of the naphthyl groups around the Si–C bonds in solution.

Figure 9 shows the frontier molecular orbitals of **24**, **25a**, and **25b** afforded by the DFT computations at the B3LYP/6-31G** level [82]. While the HOMOs mainly consist of the π(Si–Si) orbital, the LUMOs involve the appreciable contribution of the π*(Si–Si)–π*(naphthalene) conjugation. The HOMO and LUMO energy levels of **24** (–4.209 and –1.572 eV) are somewhat higher and lower than those of **25a** (–4.249 and –1.554 eV) and **25b** (–4.304 and –1.478 eV). Accordingly, the HOMO–LUMO energy gap for **24** (2.637 eV) is slightly smaller than those of **25a** (2.695 eV) and **25b** (2.826 eV). These calculations are in good qualitative agreement with the experimental data, a slightly longer absorption maximum (*λ*_max_(abs)) for **24** (521 nm) relative to **25** (504 nm), which are based on the fact that the larger HOMO and LUMO lobes are at the 1-position than at the 2-position of the naphthalene ring.

The resulting 1,2-dinaphthyldisilenes **24** and **25** also exhibit a high thermal stability with a decomposition point of 245–248 °C for **24** and 282–285 °C for **25** under an argon atmosphere. The exceptional air and thermal stabilities would open up new opportunities for application in a range of organic electronic devices, since the Si=Si unit possesses a narrower HOMO–LUMO energy gap than the C=C unit [12–22].

Actually, as shown in Figure 10, we have found that the disilene **25** can emit light in an organic light-emitting diode (OLED) [94]. To the best of our knowledge, this is the first demonstration of electroluminescence (EL) from a disilene compound in OLEDs. A typical multi-thin-layer pattern has been used for the OLEDs, in which three organic molecular layers, i.e*.* poly(3,4-ethylenedioxythiophene)-poly(styrenesulfonate) (PEDOT-PSS) [95] as the hole-injecting layer, the disilene **25** with a host molecule of poly(9,9-dioctylfluorene) (PFO) in the weight ratio of 1:1 as the light-emitting and hole transporting layer, and 2,2′,2′′-(1,3,5-benzinetriyl)-tris(1-phenyl-1-H-benzimidazole) (TPBi) as the hole-blocking and electron-transporting layer, are sandwiched between a transparent ITO (indium tin oxide) anode and metallic LiF/Al cathode on a glass substrate. This device emits a bright orange light from the disilene **25** at the applied voltage of 5 V.

Although the total performance is rather low and still far from a practical application (the maximum brightness of *L*_max_ = 119 cd m^–2^ at the driving voltage of 8.5 V, the luminance efficiency *η*_100_ and the current efficiency *L*/*J*_100_ at a luminance of 100 cd m^–2^ of 0.013 lm W^–1^ and 0.035 cd A^–1^, the external quantum efficiency (EQE) of 0.014%, and the half-life of 76.5 min of the orange light emission at a 25 mA cm^–2^ current density with the initial light output of 14.6 cd m^–2^), this study provides a new avenue for investigations to explore the applied chemistry of unsaturated compounds of the heavier main group elements. The air-stable, emissive 1,2-dinaphthyldisilenes **24** and **25** are now commercially available [96].

## 4. Disilene π-system with pyrenyl groups; evidence for intramolecular charge-transfer emission

**Scheme 6.**
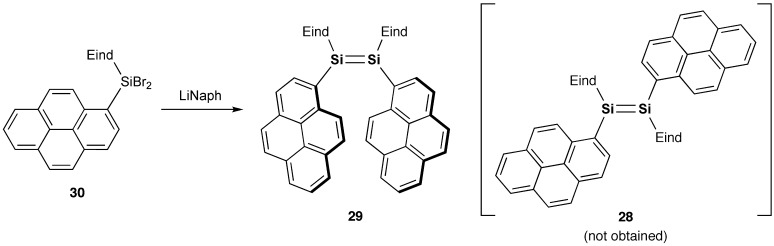
Synthesis of compound **29**.

Following the successful achievements of the (*E*)-1,2-dinaphthyldisilenes **24** and **25**, we focused on the development of further π-extended disilene molecules. For example, (*E*)-1,2-di(1-pyrenyl)disilene (**28**) can be considered as a fascinating π-system with two π-extended pyrenyl groups consisting of four fused benzene rings. As shown in Scheme 6, we have examined the reductive treatment of the Eind- and 1-pyrenyl-substituted dibromosilane, (Eind)(1-pyrenyl)SiBr_2_ (**30**), with a sufficient amount of lithium naphthalenide (LiNaph) in THF. However, unexpectedly, we obtained the *Z* isomer, (*Z*)-1,2-di(1-pyrenyl)disilene (**29**), as purple crystals in 43% yield [97]. The disilene **29** is not very stable in the air and even in the solid state, which is in sharp contrast to the fact that the red crystals of (*E*)-1,2-dinaphthyldisilenes **24** and **25** can survive in the air for more than several years. Although the formation mechanism of **29** is not yet clear, this is the first selective formation of the *Z* isomer of the acyclic disilene by the reductive coupling of monosilane precursors. The attractive π–π interaction between the pyrenyl groups may play a role in determining the stereochemistry during in the Si–Si bond-forming processes.

As shown in Figure 11, the molecular structure of **29** has been unambiguously characterized by X-ray crystallography to adopt a *Z*-configuration. The two Eind groups and the two 1-pyrenyl groups mesh in a gear-like fashion centering around the disilene core with the Si=Si bond length of 2.1718(6) Å and the *trans*-bent angles (*θ*) of 8.22(8) and 2.96(8)° (Table 1). The two pyrene rings are twisted about the Si=Si unit with the Si–Si–C–C torsion angles of 52.66(13)° and 48.73(14)°, which intramolecularly interact with each other to have a π–π stacking with a distance between the centers of the two pyrene rings of 3.635 Å.

The photophysical data of **29** are summarized in Table 2. In the UV–vis spectrum of **29** in THF, two broad absorption bands are observed with the absorption maxima (*λ*_max_(abs)) at 519 and 575 nm, together with a strong absorption around 350 nm due to the pyrene ring itself. The *λ*_max_(abs) values are found to be not sensitive to the solvent polarity. In contrast, as shown in Figure 12, the emission maximum (*λ*_max_(ex)) of **29** is dependent on the solvent polarity and red-shifted from 661 nm in hexane to 676 nm in THF and to 694 nm in acetone (dielectric constant: hexane 1.88, THF 7.58, acetone 20.56) [98]. These data indicate the intramolecular charge-transfer (ICT) emission at room temperature, which may originate from the arrangement of the two 1-pyrenyl groups twisted from the Si=Si double bond.

The photophysical properties of **29** are theoretically supported by DFT studies including excited-state calculations [97]. Figure 13 shows the three pertinent molecular orbitals of **29**. Although the HOMO is represented by the π(Si–Si) orbital, the LUMO involves a substantial π*(Si–Si)–π*(pyrene) conjugation. The LUMO+1 corresponds to the π*(pyrene) orbital. The natural population analysis (NPA) charge distribution [99] exhibits a more charge-separated character for the (*Z*)-1,2-di(1-pyrenyl)disilene skeleton in the excited state compared to the ground state, which is consistent with the π(Si–Si) → π*(pyrene) ICT excited state based on the electron transfer from the disilene π-donor toward the pyrene π-acceptor.

As shown in Figure 14, in regard to the ICT behavior of the disilenes, a unique ICT absorption by 9-anthryl-substituted trialkyldisilene (**31**) has been found by Iwamoto, Kira, and co-workers, in which the anthracene ring is orthogonal to the Si=Si unit [100]. The disilene **31** can be obtained as blue-purple crystals by the reaction of the corresponding trialkyldisilenide with 9-bromoanthracene. Iwamoto et al. also very recently reported a series of heteroaryl-substituted disilenes (**32a–c** and **33a–c**), where heteroaryl groups serve as electron acceptors for Si=Si double bonds in ICT transitions [101]. These disilenes can also be prepared by the treatment of the corresponding dialkylaryldisilenide with heteroaryl halides and dihalides. In the crystal structures of **32a**, **32b**, **33b**, and **33c**, the heteroaryl groups are almost perpendicular to the Si=Si double bond mainly due to the steric repulsion between the bulky cyclic alkyl substituents, mesityl group, and the heteroaryl groups.

Very recently, Scheschkewitz et al. also reported the photophysical properties of some related (oligo)aromatic species having one or two Si=Si double bonds (**34a–d** and **35a–d**), which have been prepared by the reaction of the triaryldisilenide **12** with aryl halides and dihalides [102]. The tetrasiladienes **35b–d** exhibit a fluorescence at room temperature. DFT calculations suggest the partial CT character of the excited state. It is important to note that the 9,10-anthracene-bridged tetrasiladiene **35d** is the first example of a near-infrared emissive disilene compound ((*λ*_max_(ex)) = 816 nm).

In order to obtain the initial target molecule, (*E*)-1,2-di(1-pyrenyl)disilene **28**, we examined the photoreaction of **29** [103]. As shown in Figure 16, after the photolysis (*λ* = 530 nm) of **29** in THF at room temperature, the solution color has changed from purple to blue. In the UV–vis absorption spectrum, a relatively new sharp peak appeared around at 590 nm, which is 15 nm red-shifted from that of **29** (575 nm), with a higher molar extinction coefficient (*ε* = 1.3 × 10^4^ cm^–1^ M^–1^) relative to that of **29** (*ε* = 7.2 × 10^3^ cm^–1^ M^–1^), thus indicating the formation of a more π-extended system. The absorption spectral change with an isosbestic point at 540 nm suggests a clean photoisomerization process from (*Z*)-**29** to (*E*)-**28**. Unfortunately, the blue **28** has not yet been isolated in a pure form due to its labile nature, but the DFT calculations indicate the highly coplanar (*E*)-1,2-di(1-pyrenyl)disilene skeleton induced by the orthogonal arrangement of the Eind groups [90]. In order to confirm the formation of **28**, we are now investigating on alternative synthetic route using the Eind-substituted (*E*)-1,2-dibromosilane, (Eind)BrSi=SiBr(Eind), as a precursor [54].

## 5. Disilene–thiophene π-systems; future organosilicon chemistry for developing advanced materials

As already described, the construction of novel π-conjugated disilene systems consisting of the carbon-based 2*p*π and silicon-based 3*p*π*-*electrons would provide exciting opportunities to explore new organosilicon chemistry for organic–inorganic hybrid materials, since the Si=Si units have a narrower HOMO–LUMO gap compared to the C=C counterparts [12–22]. Very recently, we studied a new type of π-conjugation between the Si=Si double bond and aromatic heterocycles. As shown in Figure 17, we previously examined the synthesis and electronic properties of a series of oligothiophenes (**36a**–**c**) with the bulky EMind (R^1^ = R^2^ = Et, R^3^ = R^4^ = Me) groups [50]. The orthogonal orientation of the EMind groups has proven to be useful to produce a coplanar arrangement of oligothiophene backbones. Based on these fundamental investigations, we have set out to examine the possibility to construct disilene–thiophene π-conjugated systems by the introduction of the Rind groups.

**Scheme 7.**
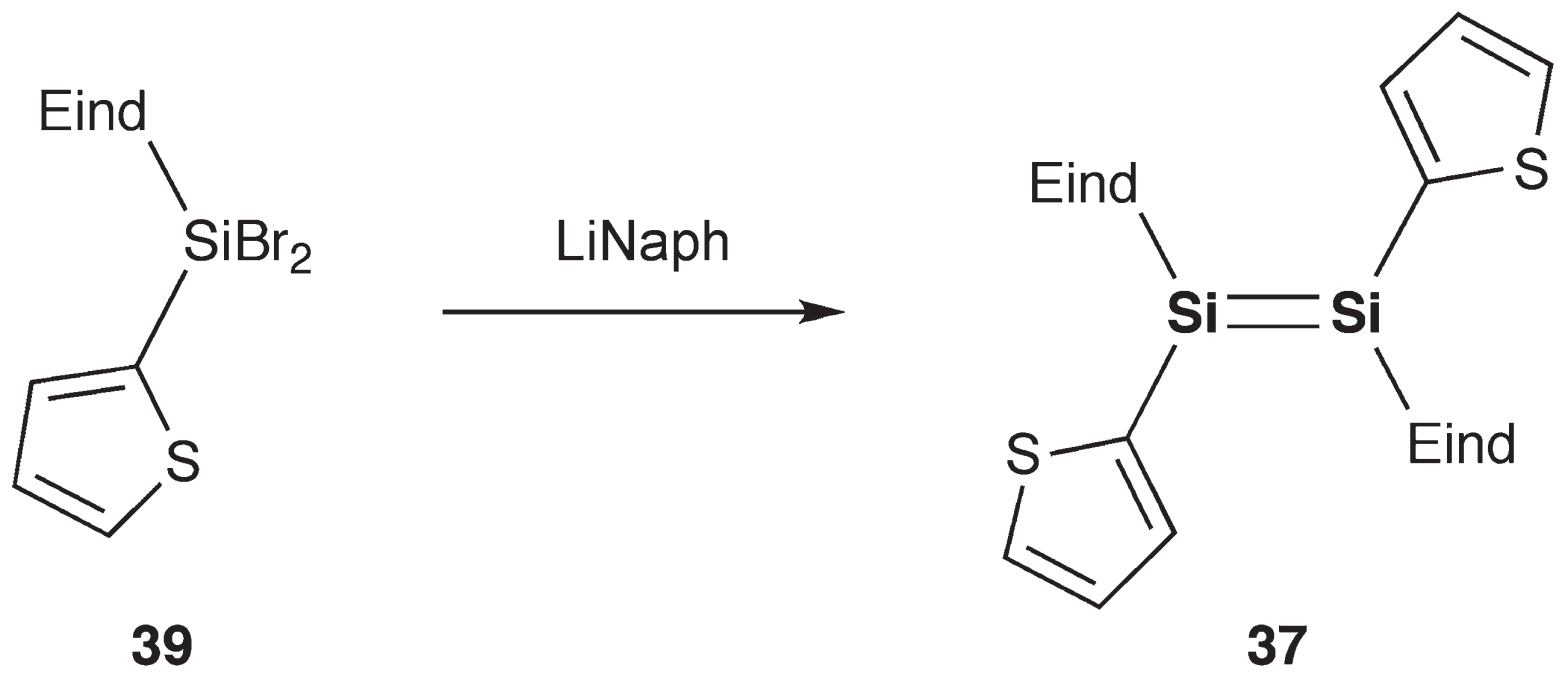
Synthesis of compound **37**.

**Scheme 8.**
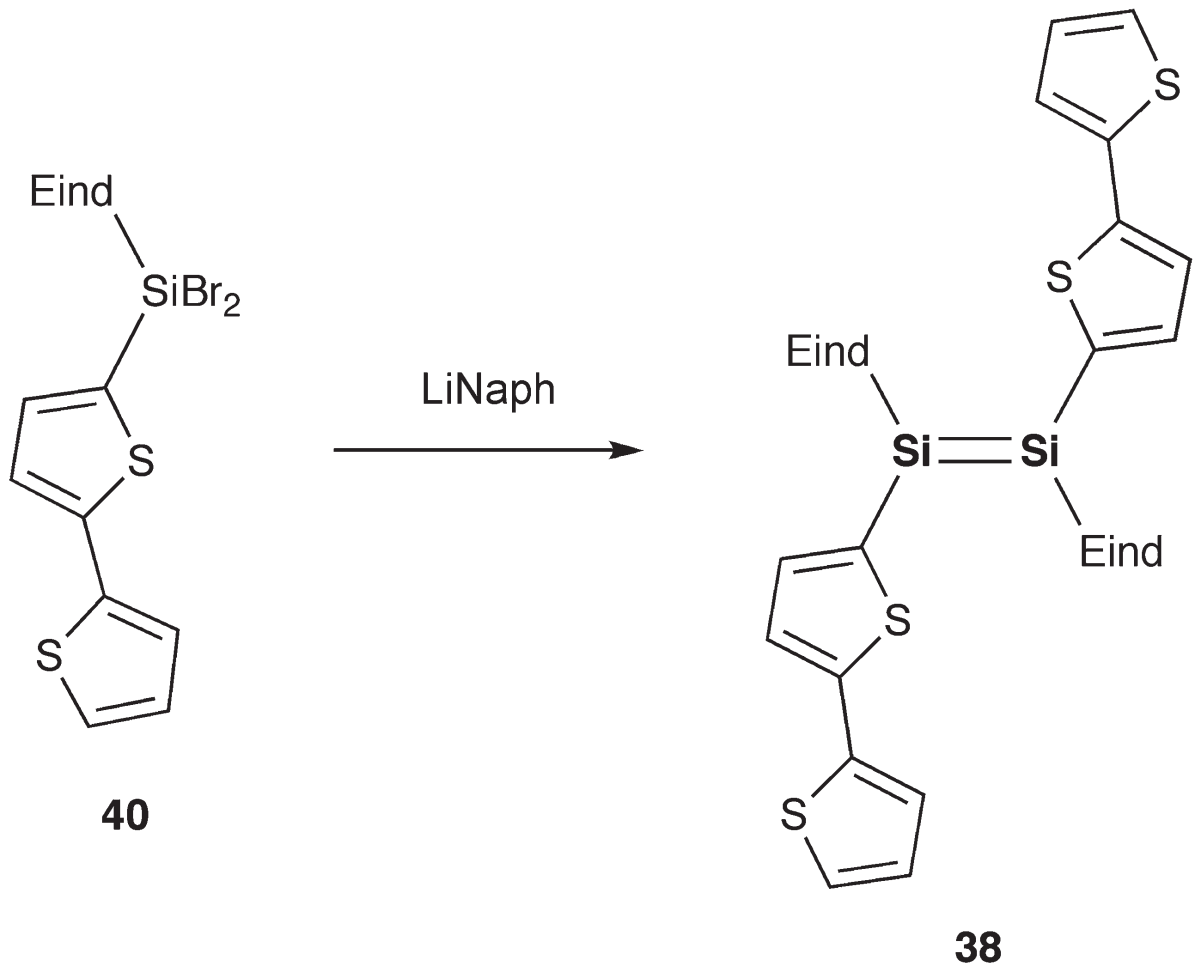
Synthesis of compound **38**.

As shown in Schemes 7 and 8, we have designed and synthesized two new disilenes, 1,2-bis(thiophen-2-yl)disilene (**37**) and 1,2-bis(2,2′-bithiophen-5-yl)disilene (**38**), as model compounds of the disilene–thiophene copolymers [86]. The disilenes **37** and **38** can be isolated as orange and purple crystals, respectively, by the reduction of the corresponding thienyl- and bithienyl-substituted dibromosilanes (**39** and **40**).

Figure 18 shows the X-ray molecular structures of **37** and **38**. Each molecule has an inversion center at the middle of the Si=Si double bond with an *E* configuration. As shown in Figure 19, there were found several rotational isomers of **37** and **38** in the crystals. For **37**, the thiophene units are disordered over the two positions with the occupancy factors of *ca*. 0.90/0.10, which is consistent with the existence of a mixture of three rotational isomers, *s*-*cis*, *s*-*cis* (**37a**), *s*-*cis*, *s*-*trans* (**37b**), and *s*-*trans*, *s*-*trans* (**37c**), with the occupancy factors of *ca*. 0.81/0.18/0.01. For **38**, while the inner thiophene rings are ordered in the crystal with an *s*-*trans*, *s*-*trans* conformation, the outer thiophene rings are disordered over the two orientations with the occupancy factors of *ca*. 0.76/0.24. Thus, the three rotational isomers, *anti*-*(s*-*trans, s*-*trans)*-*anti* (**38a**), *syn*-*(s*-*trans, s*-*trans)*-*anti* (**38b**), and *syn*-*(s*-*trans, s*-*trans)*-*syn* (**38c**), exist in the crystal with the ratio of *ca*. 0.58/0.36/0.06. It is worth mentioning that all the NMR data for **37** and **38** indicate the free-rotation around the Si–C bonds and the exocyclic C–C bonds in solution at room temperature on the NMR time scale.

The major structural parameters of **37** and **38** are summarized in Table 1. The disilene **37** has a *trans*-bent structure with the *trans*-bent angles (*θ*) of 19.12(12)° for **37a** and **37b** and 13.5(6)° for **37b** and **37c**. The Si atoms assume a somewhat pyramidal geometry with the sum of the bond angles around the Si atom (ΣSi) of *ca*. 355.5–357.5°. The disilene core of **38** exhibits a more coplanar arrangement relative to **37** with the *trans*-bent angle (*θ*) of 5.44(10)°. The Si atoms have an almost planar geometry; the sum of the bond angles around the Si atom (ΣSi) is 359.7°. The bithiophene moieties in **38** are slightly twisted with the dihedral angles between the inner and outer thiophene rings of 21.0(3)° for **38a** and **38b** and 28.6(10)° for **38b** and **38c**. The Si=Si double bond lengths of 2.1712(11) Å for **37** and 2.1584(9) Å for **38** are in the range of those for typical disilenes [16].

The photophysical data of **37** and **38** are summarized in Table 2. As shown in Figure 20, the absorption color clearly changes from the yellow of **37** to the red-purple of **38**. In the UV–vis spectrum of **37** in THF, the absorption maximum (*λ*_max_(abs)) appears at 459 nm, comparable to that of **10** (461 nm), which indicates the efficient π-conjugation between the Si=Si double bond and the two thiophene moieties. For **38**, the absorption peak is observed at 530 nm, which is 71 nm red-shifted from that of **37** (459 nm) and similar to that of **11** (543 nm). This large bathochromic shift is most likely interpreted in terms of the extension of the π-conjugation with the increasing number of thiophene units. In addition, the *λ*_max_(abs) value of **38** is 116 nm longer than that of the EMind-substituted quaterthiophene **36a** (414 nm in CH_2_Cl_2_) [50]. Thus, the insertion of the Si=Si double bond into the quaterthiophene skeleton causes a considerable narrowing of the HOMO–LUMO gap.

As shown in Figure 20, the π-extended disilene **38** displays a weak but distinct emission both in solution and in the solid state, while the disilene **37** does not show any emission at room temperature. The emission maximum (*λ*_max_(ex)) of **38** appears at 688 nm in THF with the quantum yield (Φ_F_) of 0.01. The Stokes shift (Δ*ν*_Stokes_) of **38** (4330 cm^–1^) is more than twice as high as that of **11** (2080 cm^–1^). These emission properties in solution may be explained by the structural flexibility of the 1,2-bis(bithienyl)disilene skeleton compared to the 1,4-bis(disilenyl)benzene skeleton. The disilene **38** exhibits a relatively strong emission at 691 nm in the solid state with the quantum yield (Φ_F_) of 0.11, which is about 10 times stronger than that in solution. The weaker emission in solution is attributable to the free-rotation of the bithienyl groups around the Si–C bonds and the exocyclic C–C bonds as observed in the ^1^H NMR spectrum.

In order to further clarify the structural and electronic properties of the disilenes **37** and **38**, we performed DFT calculations at the B3LYP-D3/6-31G(d,p) level [82]. The DFT studies indicated a rather flexible geometry around the disilene core in **37**. Thus, the optimized structures of **37a** (*C*_i_ symmetry), **37b** (*C*_s_ symmetry), and **37c** (*C*_1_ symmetry) exhibit an entirely coplanar 1,2-dithienyldisilene skeleton (*θ* = 0.0–0.1°), which are different from the X-ray structures (*θ* = 19.12(12)° and 13.5(6)°) found in the crystal. These rotational isomers have almost the same energies with the relative energies of 0.00 (**37a**), 1.12 (**37b**), and 2.48 (**37c**) kcal mol^–1^. The optimized structure of **38a** (*C*_1_ symmetry) shows a slightly more *trans*-bent configuration (*θ* = 10.8°) relative to that found in the crystal (*θ* = 5.44(10)°). The dihedral angles between the inner and outer thiophene rings are estimated to be 17.8°, which are somewhat smaller than those of the experimental X-ray values (21.0(3)° and 28.6(10)°). The differences between the X-ray and DFT structures are mainly due to the flexibility of the main chains consisting of the Si=Si unit and the thiophene rings, which would be easily affected by the crystal packing forces.

The molecular orbitals of **37a** and **38a** are depicted in Figure 21 in which the HOMOs mainly consist of the π(Si–Si) orbital along with a small contribution of the π(thiophene) and π(bithiophene) orbitals, while the LUMOs delocalize over the entire 1,2-dithienyldisilene and 1,2-bis(bithienyl)disilene frameworks. The HOMO level of **38a** (–4.211 eV) is comparable to that of **37a** (–4.222 eV), while the LUMO level of **38a** (–1.681 eV) is much lower than that of **37a** (–1.350 eV) due to the extended π*(Si–Si)–π*(bithiophene) conjugation. The HOMO–LUMO energy gap for **38a** (2.530 eV) is smaller than that for **37a** (2.871 eV), which are in good agreement with the UV–vis absorption data. The TD-DFT calculations almost reproduce the absorption spectra with the absorption wavelengths at 466 nm for **37a** and 558 nm for **38a**, which are comparable to those observed for **37** (459 nm) and **38** (530 nm), assignable to the HOMO → LUMO (π–π*) transitions.

In this study, we have demonstrated for the first time that the Si=Si double bond can fully conjugate with aromatic heterocycles using the appropriate steric effects due to the bulky Eind groups. The experimental and theoretical studies provide clear evidence for the effective π-conjugation between the Si=Si chromophore and thiophene units, originating from the essentially coplanar (bi)thiophene–Si=Si–(bi)thiophene skeletons. Further studies to develop promising disilene–thiophene copolymers for future application in a range of organic electronic devices are now in progress.

## 6. Concluding remarks

In this review, we have addressed the recent progress related to the chemistry of π-electron systems containing Si=Si double bonds. Especially, the unique steric effects of the fused-ring bulky Rind groups enabled us to isolate a series of structurally well-defined discrete π-conjugated disilene molecules, which exhibit an efficient delocalization of 2*p*π- and 3*p*π-electrons over the skeletons. In other words, the properly designed bulky protecting groups play pivotal roles in the structural determination and in the control of the electronic properties in the π-electron systems consisting of the carbon π-systems and Si=Si double bond(s). We hope that the present studies will provide a future challenge of pure and applied organoelement chemistry toward advanced materials science and technology.

As shown in Figure 22, after the achievement of the first persila[*n*]annulene compound, i.e. tetrasilacyclobutadiene (*n* = 4) (**7**) [34], extensive efforts have been devoted to the design and synthesis of hexasilabenzene (*n* = 6) (**41**) with a cyclic system consisting of the six formally *sp* ^2^-hybridized silicon atoms. The hexasilabenzene **41** can be regarded as the smallest fragment for silicene [1–3], the silicon analog of graphene with a two-dimensional honeycomb structure. Although some related hexasilabenzene isomers (**5**, **42**, and **43**) were synthesized as a stable crystalline compound [32,104,105], the long-considered hexasilabenzene **41** has not been isolated and remains elusive. In particular, novel electronic and optical properties as well as exotic silicon aromaticity arising from the six 3*p*π*-*electrons in **41** have attracted much attention from both experimentalists and theoreticians [106].

Also, as shown in Figure 23, a silicon analog of the linear polyacetylene, i.e*.* polysilyne (**44**), with repeating disilyne (RSi≡SiR) units, has yet to be achieved and remains as a dream compound for chemists, which would provide new opportunities for application in a range of electronic devices, because the Si=Si units have a smaller HOMO–LUMO energy gap compared to the C=C units. Some silicon analogs of 1,3-butadiene, i.e*.* the tetrasila-1,3-butadienes (**45**–**47**), have been obtained employing the bulky substituents [107–109]. We hope that further progress will be made in the construction and functions of the cyclic and linear π-electron systems involving the Si=Si double bonds.

## Disclosure statement

No potential conflict of interest was reported by the authors.

## Funding

This research is partially supported by the Precursory Research for Embryonic Science and Technology (PRESTO) from Japan Science and Technology Agency (JST); Ministry of Education, Culture, Sports, Science and Technology (MEXT) of Japan for Scientific Research on Innovative Areas, ‘Stimuli-responsive Chemical Species for the Creation of Functional Molecules’ [#2408] [grant number 24109003]; Scientific Research (B) [grant numbers 24350031, 15H03788]; MEXT-Supported Program for the Strategic Research Foundation at Private Universities 2014–2018 subsidy from MEXT and Kindai University. N.H. acknowledges the support by a Grant-in-Aid for JSPS Fellows from the Japan Society for the Promotion of Science (JSPS) [grant number JP16J01036].
